# Group I pharmaceuticals of IARC and associated cancer risks: systematic review and meta-analysis

**DOI:** 10.1038/s41598-023-50602-6

**Published:** 2024-01-03

**Authors:** Woojin Lim, Sungji Moon, Na Rae Lee, Ho Gyun Shin, Su-Yeon Yu, Jung Eun Lee, Inah Kim, Kwang-Pil Ko, Sue K. Park

**Affiliations:** 1 Department of Preventive Medicine, Seoul National University College of Medicine, Seoul, 03080 Republic of Korea; 2 Cancer Research Institute, Seoul National University, Seoul, 03080 Republic of Korea; 3 Department of Biomedical Sciences, Seoul National University Graduate School, Seoul, 03080 Republic of Korea; 4 Interdisciplinary Program in Cancer Biology, Seoul National University College of Medicine, Seoul, 03080 Republic of Korea; 5 National Evidence-based Healthcare Collaborating Agency (NECA), Seoul, 04933 Republic of Korea; 6 Department of Food and Nutrition, Seoul National University College of Human Ecology, Seoul, 08826 Republic of Korea; 7 Department of Occupational and Environmental Medicine, Hanyang University College of Medicine, Seoul, 04763 Republic of Korea; 8 Clinical Preventive Medicine Center, Seoul National University Bundang Hospital, Seongnam-si, Gyeonggi-do 13620 Republic of Korea; 9 Integrated Major in Innovative Medical Science, Seoul National University College of Medicine, Seoul, 03080 Republic of Korea

**Keywords:** Drug safety, Cancer prevention, Cancer epidemiology, Cancer prevention, Cancer epidemiology

## Abstract

We aimed to summarize the cancer risk among patients with indication of group I pharmaceuticals as stated in monographs presented by the International Agency for Research on Cancer working groups. Following the PRISMA guidelines, a comprehensive literature search was conducted using the PubMed database. Pharmaceuticals with few studies on cancer risk were identified in systematic reviews; those with two or more studies were subjected to meta-analysis. For the meta-analysis, a random-effects model was used to calculate the summary relative risks (SRRs) and 95% confidence intervals (95% CIs). Heterogeneity across studies was presented using the Higgins I square value from Cochran’s Q test. Among the 12 group I pharmaceuticals selected, three involved a single study [etoposide, thiotepa, and mustargen + oncovin + procarbazine + prednisone (MOPP)], seven had two or more studies [busulfan, cyclosporine, azathioprine, cyclophosphamide, methoxsalen + ultraviolet (UV) radiation therapy, melphalan, and chlorambucil], and two did not have any studies [etoposide + bleomycin + cisplatin and treosulfan]. Cyclosporine and azathioprine reported increased skin cancer risk (SRR = 1.32, 95% CI 1.07–1.62; SRR = 1.56, 95% CI 1.25–1.93) compared to non-use. Cyclophosphamide increased bladder and hematologic cancer risk (SRR = 2.87, 95% CI 1.32–6.23; SRR = 2.43, 95% CI 1.65–3.58). Busulfan increased hematologic cancer risk (SRR = 6.71, 95% CI 2.49–18.08); melphalan was associated with hematologic cancer (SRR = 4.43, 95% CI 1.30–15.15). In the systematic review, methoxsalen + UV and MOPP were associated with an increased risk of skin and lung cancer, respectively. Our results can enhance persistent surveillance of group I pharmaceutical use, establish novel clinical strategies for patients with indications, and provide evidence for re-categorizing current group I pharmaceuticals into other groups.

## Introduction

The International Agency for Research on Cancer (IARC) is an intergovernmental agency affiliated with the World Health Organization (WHO). The role of IARC is to conduct and coordinate research on the causes of cancer. In 1970, the IARC review committee recommended that expert groups of the IARC identify carcinogenic hazards based on a qualitative assessment of animal and human evidence. Hence, the IARC monographs program was launched to identify carcinogenic hazards and evaluate environmental causes of cancer in humans. The IARC working groups classified agents, mixtures, and exposures into one of four categories: group I, which are carcinogenic to humans, group IIA which are probably carcinogenic to humans, group IIB which are possibly carcinogenic to humans, and group III, wherein the level of carcinogenicity to humans is unclassifiable. The carcinogenic potential of many pharmaceuticals has been reviewed since 1975, and 24 pharmaceuticals were classified as group I as of 2021^1–6^.

Group I pharmaceuticals, which have sufficient evidence underpinning their carcinogenic effect in humans, are usually antineoplastic or immunosuppressive drugs used in combined regimens, while some are essential medicines designated by the WHO. However, only a few alternative drugs are available in the market, and the absence of new regimens makes it inevitable for patients with indications to continue taking these pharmaceuticals worldwide. In addition, group I immunomodulating agents are mostly used as a first-line therapy for patients with various autoimmune diseases or solid organ transplant recipients. The carcinogenicity of these agents was consistently evaluated and published as monographs by the IARC working group, and the results imply an association between pharmaceuticals and an increase in cancer risk^1–3,7^.

To our knowledge, studies using group I pharmaceuticals as risk factors and presenting summary effect sizes of associated cancer risk have not been previously published. In this context, to quantify and evaluate the cancer risk among patients with indications of group I pharmaceuticals, our study aimed to present the actual risk of cancer through a systematic review and meta-analysis. By presenting our meta-analysis results by subgroups, we aimed to enhance the persistent global surveillance of group I pharmaceutical use.

## Methods

### Group I pharmaceuticals

All group I pharmaceuticals from each monograph on pharmaceuticals and drugs were selected^1–6^. Of the total 24 group I pharmaceuticals, five pharmaceuticals, including diethylstilbestrol, chlornaphazine, phenacetine, a mixture containing phenacetine, and semustine (methyl-CCNU) have already been banned in the global drug market or removed for further investigation of their carcinogenicity^8^ Four hormone-related pharmaceuticals (combined estrogen-progestogen menopausal therapy, combined estrogen-progestogen oral contraceptives, tamoxifen, and postmenopausal estrogen therapy) were excluded since separate monographs specifically presented the carcinogenicity of hormone-related exposures. In addition, to assess the risk of artificial pharmaceuticals only, three herbal medicines (aristolochic acid, plants in contact with aristolochic acid, and opium) were excluded. After excluding 12 pharmaceuticals, cyclosporine, azathioprine, cyclophosphamide, busulfan, methoxsalen with ultraviolet (UV) radiation therapy, melphalan, chlorambucil, thiotepa, treosulfan, mustargen + oncovin + procarbazine + prednisone (MOPP), etoposide + bleomycin + cisplatin (BEP), and etoposide were included in this study.

### Inclusion criteria

This systematic review included studies that met specific PICOTS-SD (population, intervention, comparison, outcome, time, setting, study design) criteria (Supplementary Table 1)^9^ The study population included patients with indications for group I pharmaceuticals. Indications were diseases with sufficient evidence from the IARC monographs (Supplementary Table 2)^1–4,7,10,11^ The intervention was exposure (ever use) to group I pharmaceuticals. Comparisons were made to populations without exposure (never use) to group I pharmaceuticals. The outcomes were cancer sites with sufficient evidence from the IARC monographs (Supplementary Table 2)^1–4,7,10,11^. The outcome cancers suggested by the IARC for carcinogenicity in humans in association with group I pharmaceuticals included skin, hematologic, urinary bladder, and lung cancers. In this study, skin cancer included all melanoma, non-melanoma, and other skin cancers, while hematologic cancer included all types of hematologic malignancies, with lymphoma and leukemia as outcomes. The International Classification of Diseases, 10th Revision, Clinical Modification (ICD-10-CM) code was used to identify cancer outcomes in all published studies. Skin cancers were defined using ICD-10 codes C43–C44, hematologic cancers were C81–C96, urinary bladder cancer was C67, and lung cancer was C34. The study period was between January 1st, 1990 and December 31st, 2021. All settings were epidemiological research settings. The study design was observational, including cohort and case–control studies as non-randomized studies and randomized controlled trials (RCT) as randomized studies (Supplementary Table 1). In addition, for a study to be included in the systematic review, it had to meet the following inclusion criteria: (1) the exposed group must have received cyclosporine, azathioprine, cyclophosphamide, busulfan, melphalan, methoxsalen + UV, chlorambucil, MOPP, BEP, etoposide, thiotepa, or treosulfan treatment; (2) the study must have been specifically designed to evaluate cancer as an adverse outcome of intervention; (3) the subjects of the study must have been limited to humans; (4) the original article published in English or Korean must have presented the relative risk (RR), hazard ratio (HR), odds ratio (OR), or incidence rate ratio (IRR); and (5) studies must have only used patients with an indication as the study population for each group I pharmaceutical. Studies that did not meet these criteria were excluded.

### Search strategies

To conduct a systematic review, search terms for each group I pharmaceutical were used in the database to identify studies that met the inclusion criteria^12–14^. Specifically, both PubMed (MEDLINE) and Embase were used as search databases for comprehensive literature search. Keywords were used with reference to Medical Subject Headings (MeSH) terms to increase the sensitivity of the search strategy in PubMed (Supplementary Table 3)^15^. The online search was conducted on September 24, 2021, and was limited to studies published between 1990 and 2021 in English or Korean. In addition, to update our systematic review up to date, we have conducted additional search to find studies published from September 24, 2021, to October 31, 2023, to additionally include studies published after the period defined in this study.

Studies were identified from the database according to the PRISMA 2020 flow diagram for systematic reviews^16^. Records were screened and reports were sought for retrieval. The remaining reports were assessed for eligibility and were included in the final review. The included studies were fully reviewed by two authors, and disagreements were resolved via discussion and further review. For data extraction, the information on intervention, outcome, indication, study design, first author, publication year, study region, study period, number, and age of the study participants, effect size, and data on matched and adjusted variables from each study were extracted using EndNote 21 and by directly reviewing the text of each literature. The extracted data from each study were then organized by separate tables according to each group-I pharmaceuticals (Supplementary Tables 4–7).

The PRISMA 2020 checklist of the systematic review is presented in Supplementary Table 8.

### Statistical analysis

To calculate a summary estimate of the incidence of each cancer type, all studies had to be analyzed in the same manner. The summary relative risk (SRR) for each group I pharmaceutical and associated cancer type was determined using a random-effects model owing to the presence of significant heterogeneity^12,17^ The heterogeneity between included studies was calculated using Cochran’s Q test and presented as Higgins I square (%) value^18^. An I^2^ value below 34% (I^2^ < 34) was considered as low heterogeneity, an I^2^ value between 34% and 67% (34 ≤ I^2^ < 67) was considered as intermediate heterogeneity, and an I^2^ square value over 67% (I^2^ ≥ 67) was considered as high heterogeneity. The publication bias of the included studies was assessed using the Begg and Egger test. Subgroup analyses were performed according to the type of indication, study design, publication period, and study region to determine the reasons for possible heterogeneity. Significant heterogeneity was defined as *P* < 0.05.

All meta-analyses were performed using the “meta” R package^19^. For the quality assessment of studies included in the meta-analysis, the Risk of Bias Assessment tool for Non-randomized Studies (RoBANS) and Cochrane’s Risk of Bias (RoB) tool were used to assess the individual risk of bias in non-randomized and randomized studies, respectively. The evaluation criteria for RoBANS included the comparability of participants, selection of participants, confounding variables, measurement of exposure, blinding of outcome assessment, outcome evaluation, incomplete outcome data, and selective reporting. The evaluation criteria for RoB included random sequence generation, allocation concealment, blinding of outcome assessment for participants and personnel, blinding of outcome assessment for outcome assessors, incomplete data, and selective reporting.

To identify the robustness of the results, sensitivity analysis was conducted. For each group-I pharmaceuticals and associated cancers, heterogeneity was calculated after omitting each individual study by conducting influential meta-analysis with random effects model. In the influential analysis, inverse variance and DerSimonian-Laird method were used to estimate *p* values, tau values and confidence intervals.

## Results

### Systematic review

To determine the association of cyclosporine with the risk of skin cancer and hematologic cancer, 13 out of 2522 studies^20–32^ and 6 out of 8444 studies^20,33–37^ were included in the final review, respectively. To determine the association of azathioprine with the risk of skin cancer and hematologic cancer, 13 out of 1854 studies^21–25,27–29,31,38–41^ and 12 out of 4027 studies^33–38,42–47^ were included in the final review, respectively. For the association of cyclophosphamide with the risk of urinary bladder cancer and hematologic cancer, 3 out of 1782 studies^48–50^ and 7 out of 50,301 studies^43,44,50–54^ were included in the final review, respectively. For the association of busulfan with the risk of hematologic cancer, 2 out of 5,131 studies^55,56^ were included in the final review. One out of 642 studies^57^ assessing the association of methoxsalen + UV with the risk of skin cancer was included in the final review. For the association of melphalan and chlorambucil with the risk of hematologic cancer, 3 out of 8358 studies^51,52,58^ and 2 out of 4965 studies^52,54^ were included in the final review, respectively. For the association of MOPP with the risk of lung cancer, 1 out of 677 studies^59^ was included in the final review. For the association of thiotepa and etoposide with the risk of hematologic cancer, 1 each out of 1883 and 4746 studies^52,60^, respectively, were included in the final review. For the association with BEP, MOPP, and treosulfan on the risk of hematologic cancer, no studies out of 63, 445, and 59 studies were included in the final review (Table 1, Supplementary Fig. 1–16). In addition, no studies published from September 24, 2021, to October 31, 2023, neither met our criteria nor were additionally included in our systematic review.

**Table 1:** Number of studies identified through the systematic review.

Group I pharmaceuticals	Outcome cancer	Identification		Screening	Included	
		Records identified from databases (n)	Records screened (n)	Reports sought for retrieval	Reports assessed for eligibility (n)	Studies included in final review (n)
Cyclosporine	Skin	2522	2522	39	39	13
	Hematologic	8444	8444	106	106	6
Cyclophosphamide	Bladder	1782	1782	20	20	3
	Hematologic	50,301	50,301	81	81	7
Azathioprine	Skin	1854	1854	31	31	13
	Hematologic	4027	4027	50	50	12
Busulfan	Hematologic	5131	5131	25	25	2
Methoxsalen + UV	Skin	642	642	25	25	1
Melphalan	Hematologic	8358	8358	30	30	3
Chlorambucil	Hematologic	4965	4965	13	13	2
Thiotepa	Hematologic	1883	1883	4	4	1
Treosulfan	Hematologic	59	59	2	2	0
MOPP	Hematologic	445	445	2	2	0
	Lung	677	677	16	16	1
BEP	Hematologic	63	63	9	9	0
Etoposide	Hematologic	4746	4746	13	13	1

*UV* ultraviolet, *MOPP* mustargen + oncovin + procarbazine + prednisone, *BEP* etoposide + bleomycin + cisplatin.

### Meta-analysis

**Figure 1:**
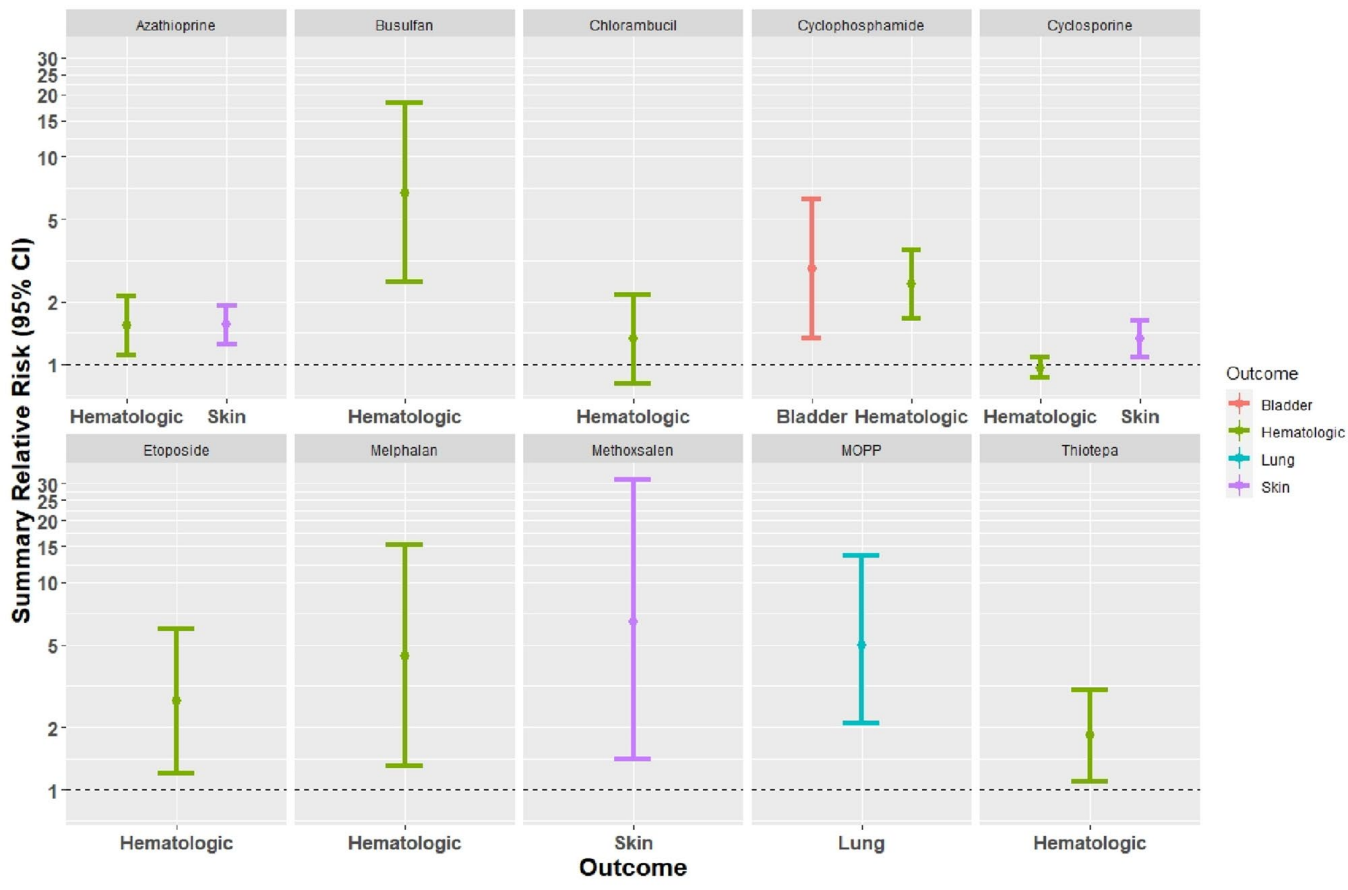
SRR (95% CI) of the risk of subsequent cancers in patients exposed to group I pharmaceuticals.

The SRR of skin cancer in association with cyclosporine and azathioprine was 1.32, with 95% CI 1.07–1.62 and 1.56, with 95% CI 1.25–1.93, respectively. The SRR of hematologic cancer in association with cyclosporine and azathioprine was 0.96, with 95% CI 0.86–1.07 and 1.53, with 95% CI 1.10–2.12, respectively. In addition, the SRR of bladder and hematologic cancer in association with cyclophosphamide was 2.87, with 95% CI 1.32–6.23 and 2.43, with 95% CI 1.65–3.58, respectively. The SRR of hematologic cancer in association with busulfan, chlorambucil and melphalan was 6.71 with 95% CI 2.49–18.08, 1.32 with 95% CI 0.81–2.16, and 4.43 with 95% CI 1.30–15.15, respectively. The SRR of skin cancer in association with methoxsalen + UV was 6.50, with 95% CI 1.40–31.40, and the SRR of lung cancer in association with MOPP was 5.00 with 95% CI 2.10–13.60. The SRR of hematologic cancer in association with etoposide and thiotepa was 2.70 with 95% CI 1.20–6.00 and 1.82 with 95% CI 1.09–3.03, respectively (Table 2, Fig. 1).

**Table 2:** SRR (95% CI) of the risk of subsequent cancers in patients exposed to group I pharmaceuticals.

Group I pharmaceuticals	Cancer outcome	Study N	Study period	SRR (95% CI)
Cyclosporine	Skin	13^20–32^	1963–2015	**1.32 (1.07–1.62)**^**H**^
	Hematologic	6^20,33–37^	1970–2015	0.96 (0.86–1.07)^L^
Azathioprine	Skin	13^21–25,27–29,31,38–41^	1960–2014	**1.56 (1.25–1.93)**^**I, E, B**^
	Hematologic	12^33–38,42–47^	1958–2011	**1.53 (1.10–2.12)**^**H, E, B**^
Cyclophosphamide	Bladder	3^48–50^	1960–2011	**2.87 (1.32–6.23)**^**I**^
	Hematologic	7^43,44,50–54^	1958–2011	**2.43 (1.65–3.58)**^**I**^
Busulfan	Hematologic	2^55,56^	1964–2001	**6.71 (2.49–18.08)**^**L**^
Chlorambucil	Hematologic	2^52,54^	1965–1989	1.32 (0.81–2.16)^L^
Methoxsalen + UV	Skin	1^57^	1973–1995	**6.50 (1.40–31.40)**
Melphalan	Hematologic	3^51,52,58^	1970–1993	**4.43 (1.30–15.15)**^**H**^
Etoposide	Hematologic	1^60^	1980–1999	**2.70 (1.20–6.00)**
Thiotepa	Hematologic	1^52^	1970–1985	**1.82 (1.09–3.03)**
MOPP	Lung	1^59^	1965–1994	**5.00 (2.10–13.60)**
	Hematologic	No studies included in the systematic review		
Treosulfan	Hematologic			
BEP	Hematologic			

*Study N* study number, *SRR* summary relative risk, *CI* confidence interval, *UV* ultraviolet, *MOPP* mustargen-oncovin-procarbazine-prednisone mixture, *BEP* bleomycin-etoposide-platinum (Cisplatin) mixture, *NHL* non-Hodgkin lymphoma, *SRR* summary relative risk, *L* low heterogeneity (I^2^ < 34), *I* intermediate heterogeneity (34 ≤ I^2^ < 67), *H* high heterogeneity (67 ≤ I^2^), *E* significant publication bias *p* < 0.05 in Egger test, *B* significant publication bias *p* < 0.05 in Begg test.

Significant values are in [bold].

In the subgroup analysis of indications, skin cancer risk was associated with cyclosporine use in patients with rheumatoid arthritis (SRR = 3.55, 95% CI 1.60–7.85). Hematologic cancer risk was associated with azathioprine use in patients with inflammatory bowel disease (IBD) (SRR = 3.77, 95% CI 2.56–5.54), while skin cancer risk was associated with azathioprine use in solid organ transplant recipients (SRR = 1.43, 95% CI 1.11–1.83) (Table 3). When subgroup analysis was conducted by study design, case–control studies showed a higher risk of skin cancer associated with azathioprine use (SRR = 2.61, 95% CI 1.35–5.05) than cohort studies (SRR = 1.32, 95% CI 1.11–1.58) (Table 4). When subgroup analysis was conducted by publication year, skin cancer risk associated with azathioprine use was similar in studies published after 2010 (SRR = 1.58, 95% CI 1.19–2.10) compared to studies published before 2010 (SRR = 1.59, 95% CI 1.07–2.38) (Table 5). When subgroup analysis was conducted by study region, increased skin cancer risk with azathioprine use was noted in studies conducted in Europe (SRR = 2.19, 95% CI 1.36–3.50), compared to studies conducted in North America (SRR = 1.27, 95% CI 1.00–1.61) (Table 6). In addition, based on the summarized result of the quality assessment of the studies, all non-randomized studies showed low risk of bias within “incomplete outcome data” and “blinding of outcome assessment” criteria of RoBANS. A single RCT study^55^ was evaluated through RoB and showed low risk of bias within “incomplete data” criteria (Supplementary Fig. 17). Among the 40 non-randomized studies, 15 studies showed a low risk of bias in all eight RoBANS evaluation criteria (Supplementary Fig. 18).

**Table 3:** SRR (95% CI) of the risk of subsequent cancers in patients exposed to group I pharmaceuticals by indication.

Group I pharmaceuticals	Cancer outcome	Indications	Study N	SRR (95% CI)
Cyclosporine	Skin	Solid organ transplant	11^20–28,30,31^	1.17 (0.98–1.40)^i^
		Rheumatoid arthritis	2^29,32^	**3.55 (1.60–7.85)**^**i**^
	Hematologic	Solid organ transplant	6^20,33–37^	0.96 (0.86–1.07)^l^
Azathioprine	Skin	Solid organ transplant	8^21–25,27,28,31^	**1.43 (1.11–1.83)**^**i**^
		Inflammatory bowel disease	3 ^38,40,41^	1.63 (0.99–2.66)^l^
		Myasthenia	1^39^	**3.30 (1.50–7.30)**
		Rheumatoid arthritis	1^29^	1.94 (0.87–4.34)
	Hematologic	Solid organ transplant	7^33–37,46,47^	1.08 (0.81–1.45)^h^
		Inflammatory bowel disease	3^38,42,45^	**3.77 (2.56–5.54)**^**l**^
		Rheumatoid arthritis	1^43^	1.07 (0.74–1.54)
		Systemic lupus erythematosus	1^44^	1.19(0.48–2.92)
Cyclophosphamide	Bladder	Non-Hodgkin lymphoma	2^49,50^	2.31 (0.58–9.24)^i^
		Ovarian cancer	1^48^	**4.20 (1.20–14.00)**
	Hematologic	Non-Hodgkin lymphoma	2^50,54^	1.59 (0.71–3.55)^l^
		Breast cancer	1^51^	**3.10 (1.30–7.70)**
		Lymphoma	1^53^	**14.80 (3.70–59.4)**
		Rheumatoid arthritis	1^43^	**1.84 (1.24–2.73)**
		Ovarian cancer	1^52^	**2.65 (1.64–4.26)**
		Systemic lupus erythematosus	1^44^	2.09 (0.69–6.30)
Busulfan	Hematologic	Essential thrombocythemia	1^55^	**4.48 (1.11–27.10)**
		Polycythemia vera	1^56^	**8.64 (2.44–30.60)**
Chlorambucil	Hematologic	Non-Hodgkin lymphoma	1^54^	2.40 (0.70–8.60)
		Ovarian cancer	1^52^	1.19 (0.72–1.97)
Methoxsalen + UV	Skin	Psoriasis	1^57^	**6.50 (1.40–31.40)**
Melphalan	Hematologic	Breast cancer	1^51^	**3.10 (1.30–7.70)**
		Ovarian cancer	2^52,58^	5.83 (0.55–61.2)^h^
MOPP	Lung	Hodgkin disease	1^59^	**5.00 (2.10–13.60)**
Etoposide	Hematologic	Solid tumor	1^60^	**2.70 (1.20–6.00)**
Thiotepa	Hematologic	Ovarian cancer	1^52^	**1.82 (1.09–3.03)**

*Study N* study number, *SRR* summary relative risk, *CI* confidence interval, *UV* ultraviolet, MOPP mustargen-oncovin-procarbazine-prednisone mixture, *l* low heterogeneity (I^2^ < 34), *i* intermediate heterogeneity (34 ≤ I^2^ < 67), *h* high heterogeneity (67 ≤ I^2^).

Significant values are in [bold].

**Table 4:** SRR (95% CI) of the risk of subsequent cancers in patients exposed to group I pharmaceuticals by study design.

Group I pharmaceuticals	Cancer outcome	Study design	Study N	SRR (95% CI)
Cyclosporine	Skin	Cohort	10^20–22,24,26–31^	**1.23 (1.00–1.51)**^**H**^
		Case–control	3^23,25,32^	2.03 (0.79–5.23)^H^
	Hematologic	Cohort	5^20,33,34,36,37^	0.94 (0.83–1.07)^L^
		Case–control	1^35^	0.90 (0.20–5.80)
Azathioprine	Skin	Cohort	9^21,22,24,27–29,31,38,40^	**1.32 (1.11–1.58)**^**L**^
		Case–control	4^23,25,39,41^	**2.61 (1.35–5.05)**^**H**^
	Hematologic	Cohort	10^33,34,36–38,42,44–47^	**1.53 (1.10–2.12)**^**H**^
		Case–control	2^35,43^	1.30 (0.61–2.78)^L^
Cyclophosphamide	Bladder	Case–control	3^48–50^	**2.87 (1.32–6.23)**^**I**^
	Hematologic	Cohort	1^44^	2.09 (0.69–6.30)
		Case–control	6^43,50–54^	**2.50 (1.61–3.90)**^**I**^
Busulfan	Hematologic	Cohort	1^56^	**8.64 (2.44–30.60)**
		RCT	1^55^	**4.48 (1.11–27.10)**
Chlorambucil	Hematologic	Case–control	2^52,54^	1.32 (0.81–2.16)^L^
Methoxsalen + UV	Skin	Case–control	1^57^	**6.50 (1.40–31.40)**
Melphalan	Hematologic	Case–control	3^51,52,58^	**4.43 (1.30–15.15)**^**H**^
MOPP	Lung	Case–control	1^59^	**5.00 (2.10–13.60)**
Etoposide	Hematologic	Case–control	1^60^	**2.70 (1.20–6.00)**
Thiotepa	Hematologic	Case–control	1^52^	**1.82 (1.09–3.03)**

*Study N* study number, *SRR* summary relative risk, *CI* confidence interval, *UV* ultraviolet, *MOPP* mustargen-oncovin-procarbazine-prednisone mixture, *RCT* randomized controlled trial, *L* low heterogeneity (I^2^ < 34), *I* intermediate heterogeneity (34 ≤ I^2^ < 67), *H* high heterogeneity (67 ≤ I^2^).

Significant values are in [bold].

**Table 5:** SRR (95% CI) of the risk of subsequent cancers in patients exposed to group I pharmaceuticals by publication year.

Group I pharmaceuticals	Cancer outcome	Publication year	Study N	SRR (95% CI)
Cyclosporine	Skin	≤ 2010	6^25–28,30,31^	**1.61 (1.08–2.42)**^**H**^
		> 2010	7^20–24,29,32^	1.25 (0.91–1.70)^H^
	Hematologic	≤ 2010	1^33^	0.80 (0.61–1.05)
		> 2010	5^20,34–37^	0.99 (0.88–1.11)^L^
Azathioprine	Skin	≤ 2010	4^25,27,28,31^	**1.59 (1.07–2.38)**^**H**^
		> 2010	9^21–24,29,38–41^	**1.58 (1.19–2.10)**^**L**^
	Hematologic	≤ 2010	5^33,42–44,47^	1.55 (0.90–2.69)^H^
		> 2010	7^34–38,45,46^	1.56 (0.93–2.61)^H^
Cyclophosphamide	Bladder	≤ 2010	2^48,49^	**4.00 (1.99–8.02)**^**L**^
		> 2010	1^50^	1.09 (0.30–3.97)
	Hematologic	≤ 2010	6^43,44,51–54^	**2.56 (1.70–3.86)**^**I**^
		> 2010	1^50^	1.20 (0.28–5.09)
Busulfan	Hematologic	≤ 2010	2^55,56^	**6.71 (2.49–18.08)**^**L**^
Chlorambucil	Hematologic	≤ 2010	2^52,54^	1.32 (0.81–2.16)^L^
Methoxsalen + UV	Skin	≤ 2010	1^57^	**6.50 (1.40–31.40)**
Melphalan	Hematologic	≤ 2010	3^51,52,58^	**4.43 (1.30–15.15)**^**H**^
MOPP	Lung	≤ 2010	1^59^	**5.00 (2.10–13.60)**
Etoposide	Hematologic	≤ 2010	1^60^	**2.70 (1.20–6.00)**
Thiotepa	Hematologic	≤ 2010	1^52^	**1.82 (1.09–3.03)**

*SRR* summary relative risk, *CI* confidence interval, *I*^*2*^ Higgin’s I square value, *P-Cochran P* value of the Cochran’s Q test, *UV* ultraviolet, *MOPP* mustargen-oncovin-procarbazine-prednisone mixture, *L* low heterogeneity (I^2^ < 34), *I* intermediate heterogeneity (34 ≤ I^2^ < 67), *H* high heterogeneity (67 ≤ I^2^).

Significant values are in [bold].

**Table 6:** SRR (95% CI) of the risk of subsequent cancers in patients exposed to group I pharmaceuticals by study region.

Group I pharmaceuticals	Cancer outcome	Study region	Study N	SRR (95% CI)
Cyclosporine	Skin	North America	6^20–23,27,30^	1.13 (0.94–1.37)^I^
		Europe	4^25,26,28,31^	1.67 (0.98–2.86)^I^
		Australia	1^29^	**2.51 (1.23–5.13)**
		Asia	1^32^	**5.70 (2.19–14.81)**
		Multicenter	1^24^	0.64 (0.38–1.08)
	Hematologic	North America	2^20,33^	0.92 (0.74–1.13)^I^
		Europe	3^34,35,37^	1.00 (0.85–1.17)^L^
		Australia	1^36^	0.73 (0.37–1.46)
Azathioprine	Skin	North America	5^21–23,27,41^	**1.27 (1.00–1.61)**^**I**^
		Europe	5^25,28,31,38,39^	**2.19 (1.36–3.50)**^**I**^
		Australia	1^29^	1.94 (0.87–4.33)
		Africa	1^40^	**5.10 (1.12–23.22)**
		Multicenter	1^24^	1.21 (0.71–2.07)
	Hematologic	North America	5^33,43–46^	1.20 (0.65–2.20)^H^
		Europe	6^34,35,37,38,42,47^	**2.06 (1.13–3.75)**^**H**^
		Australia	1^36^	**1.88 (1.03–3.42)**
Cyclophosphamide	Bladder	Asia	1^50^	1.09 (0.30–3.97)
		Multicenter	2^48,49^	**4.00 (1.99–8.02)**^**L**^
	Hematologic	North America	4^43,44,51,53^	**3.06 (1.46–6.40)**^**I**^
		Asia	1^50^	1.20 (0.28–5.03)
		Europe	1^52^	**2.65 (1.64–4.26)**
		Multicenter	1^54^	1.80 (0.70–4.90)
Busulfan	Hematologic	Europe	2^55,56^	**6.71 (2.49–18.08)**^**L**^
Chlorambucil	Hematologic	Multicenter	1^54^	2.40 (0.70–8.60)
		Europe	1^52^	1.19 (0.72–1.97)
Methoxsalen + UV	Skin	Europe	1^57^	**6.50 (1.40–31.40)**
Melphalan	Hematologic	North America	1^51^	**3.10 (1.30–7.70)**
		Europe	1^52^	**1.88 (1.33–2.65)**
		Multicenter	1^58^	**20.80 (6.30–68.30)**
MOPP	Lung	Multicenter	1^59^	**5.00 (2.10–13.60)**
Etoposide	Hematologic	Europe	1^60^	**2.70 (1.20–6.00)**
Thiotepa	Hematologic	Europe	1^52^	**1.82 (1.09–3.03)**

*Study N* study number, *SRR* summary relative risk, *CI* confidence interval, *I*^*2*^ Higgin’s I square value, *P-Cochran P* value of the Cochran’s Q test, *UV* ultraviolet, *MOPP* mustargen-oncovin-procarbazine-prednisone mixture, *L* low heterogeneity (I^2^ < 34), *I* intermediate heterogeneity (34 ≤ I^2^ < 67), *H* high heterogeneity (67 ≤ I^2^).

Significant values are in [bold].

When sensitivity analysis was conducted by influential meta-analysis, the heterogeneity of studies in association with cyclosporine and skin cancer was reduced from 72.2% to 65.8%. On the other hand, the heterogeneity of studies in association with cyclosporine and hematologic cancer remained the same (from 0% to 0%). The heterogeneity of studies in association with azathioprine and skin cancer was reduced from 57.7% to 46.5%, and the heterogeneity of studies in association with azathioprine and hematologic cancer was also reduced from 83.6% to 72.6%. In the same manner, the heterogeneity of studies in association with cyclophosphamide and bladder cancer was dramatically reduced from 35.2% to 0%, and the heterogeneity of studies in association with cyclophosphamide and hematologic cancer was also dramatically reduced from 39.8% to 0%. The heterogeneity of studies in association with melphalan and hematologic cancer was also dramatically reduced from 86.5% to 5.3% (Supplementary Table 9). In addition, the contribution of each study to the overall heterogeneity, and the standardized difference of overall SRR with and without each study was plotted for each group I pharmaceuticals and associated cancers (Supplementary Fig. 19–25).

## Discussion

Group I pharmaceuticals are mostly utilized as an essential first-line treatment for solid organ transplant recipients and immune system-related diseases. Our results indicate that the risk of skin and hematologic cancer tends to increase in patients with indications of group I pharmaceuticals. Among the group I pharmaceuticals included in this study, cyclosporine, azathioprine, cyclophosphamide, methoxsalen + UV, busulfan, and melphalan were associated with a notably significant increase in cancer risk. Specifically, an increase in skin cancer risk was confirmed in solid organ transplant recipients treated with cyclosporine, while an increase in skin and hematologic cancer risk was observed in solid organ transplant recipients and IBD patients treated with azathioprine, respectively. Moreover, an increase in bladder and hematologic cancer risks was observed in patients receiving cyclophosphamide treatment.

Busulfan is an antineoplastic agent with cell-cycle nonspecific alkylating action^4^ and is used as a palliative treatment for chronic myelogenous leukemia. Busulfan is also used for the treatment of polycythemia vera, myelofibrosis, primary thrombocythemia, and as conditioning regimens to prepare patients for stem cell transplantation^4^. The findings of the meta-analysis showed a strong association between busulfan use and hematologic cancer incidence with an over sixfold increased risk; thus, an alternative regimen is necessary for patients with polycythemia vera and essential thrombocythemia.

A 6-fold increased risk of skin cancer was also observed in patients treated with methoxsalen + UV. Methoxsalen is a photosensitizer that markedly increases skin reactivity to long-wavelength ultraviolet radiation (320–400 nm). Methoxsalen is used in photochemotherapy or psoralen (P) and high-intensity long-wavelength (UVA) irradiation (PUVA) therapy^2,4^. Methoxsalen is used in conjunction with controlled exposure to UVA radiation for the symptomatic treatment of severe, recalcitrant, and disabling psoriasis. It is also used in conjunction with photopheresis for the palliative treatment of skin manifestations of cutaneous T-cell lymphoma, chronic graft-versus-host disease, and rejection after solid organ transplant^2,4^. Nonetheless, the strong association between methoxsalen use and a 6-fold increase in skin cancer risk indicates that using methoxsalen in combination with UV irradiation should be avoided to prevent possible skin cancer in patients with psoriasis.

The results showed a nearly 3-fold increased risk of urinary bladder and hematologic cancers in patients treated with cyclophosphamide. Cyclophosphamide is an antineoplastic agent metabolized to activate alkylating metabolites^3,4^. Cyclophosphamide is also used in combination with other antineoplastic agents in treating a broad spectrum of diseases, such as chronic lymphocytic leukemia, soft tissue and osteogenic sarcoma, solid tumors, and multiple myeloma. It is also used for the treatment of Hodgkin and non-Hodgkin lymphomas, as well as high-grade lymphomas, such as Burkitt lymphoma and lymphoblastic lymphomas^3,4^. However, considering that the SRR of cyclophosphamide use indicates an almost 3-fold increased risk of urinary bladder and hematologic cancer in patients with various indications, especially cancer, the use of conventional combination chemotherapies containing cyclophosphamide as one of the main components is in question.

In general, immunosuppressants also showed a high risk, with over 50% increased risk, of skin and hematologic cancer in patients treated with azathioprine. Azathioprine is an immunosuppressant that is converted to 6-mercaptopurine after absorption^3,4^. Azathioprine is used to prevent kidney allograft rejection, manage the signs and symptoms of rheumatoid arthritis (RA) and IBD in adults, and treat acute lymphocytic leukemia in children^3,4^. Despite its various uses, an alternative prescription is required for patients with psoriasis, RA, IBD, and solid organ transplant recipients, considering the 50% increased risk of skin and hematologic cancer in patients treated with azathioprine.

Our results showed an over 30% increased risk of skin cancer in patients treated with cyclosporine, but no significant increase in risk was observed between cyclosporine use and hematologic cancer. Cyclosporine is an immunosuppressant that functions as a calcineurin inhibitor and is mainly used for the prevention of graft rejection after solid organ transplant, treatment for prophylaxis and graft-versus-host disease, and the management of the active stage of severe RA and recalcitrant plaque psoriasis^4,10^. While the SRR of cyclosporine use indicates a 30% increased risk of skin cancer, reducing the total cumulative dose and duration of use is recommended for patients with psoriasis, RA, and solid organ transplant recipients.

Notably, the SRR of skin cancer incidence associated with azathioprine and cyclosporine use in case–control studies was nearly twice as high as that in cohort studies. The interpretation of this particular difference in SRR is that among the three case–control studies on the association between cyclosporine and skin cancer, Tseng et al. used randomly sampled beneficiaries from the Taiwan National Health Insurance Research Database (NHIRD) as controls to match patients with RA^32^. In addition, among the four case–control studies on the association between azathioprine and skin cancer, Singh et al. used randomly selected controls to match patients with IBD^41^. While all study populations comprised patients with indications in cohort studies, the difference in baseline risk between azathioprine- and cyclosporine-unexposed populations from cohort studies and controls from case–control studies could explain the higher SRR in case–control studies compared to cohort studies. We could not present the SRR for methoxsalen + UV, MOPP, thiotepa, treosulfan, and etoposide because only a single study assessed the association of these agents with the risk of cancer. Moreover, no studies assessing the risk of cancer in association with treosulfan and BEP were included in the systematic review.

This study also presented the subgroup analysis results by indication, publication year, and study region. Comparisons of the subgroup analysis results between studies published before and after 2010, studies conducted in North America and Europe were possible. In addition to the study region, and publication year, we also categorized studies according to patient indications. By conducting subgroup analyses, a decrease in heterogeneity and a detailed risk assessment of each group I pharmaceutical use and associated cancer risk were possible. While most previous meta-analyses evaluated the risk of single group I pharmaceuticals as a risk factor, our study included 12 group I pharmaceuticals from IARC monographs^1–6^ as risk factors and patients with various indications as the study population. The diversity of inclusion adds robustness to our findings and makes it possible to assess and compare the risk of group I pharmaceutical use in two or more study populations with different indications.

However, our study has some limitations. First, although we conducted comprehensive systematic review, a relatively small number of studies were included in the meta-analysis. Therefore, the statistical power and robustness of the results might be undermined owing to the lack of included studies^61,62^. In addition, some cases were observed wherein the weight of a single study overpowered the total (sum) weight of the remaining studies included in the meta-analysis. Second, although we conducted subgroup analyses by study region, publication year, and indication, the heterogeneity did not decrease significantly. Third, when subgroup analysis was conducted by the study region, most of the studies were conducted in Europe and North America; only a few or no studies were conducted in Asia, Africa, or South America. Therefore, our results cannot represent cancer risk associated with group I pharmaceutical use in Asian, African, or South American populations. Finally, we could not present subgroup analysis results according to the cumulative dose and duration of use of each group I pharmaceutical as only a few studies presented risks by cumulative dose and duration of use. Hence, the risk of long-term use of group I pharmaceuticals has not yet been presented.

Through this systematic review and meta-analysis, we confirmed a significant association between azathioprine, cyclosporine, and skin cancer risk. In addition, we confirmed a strong and significant association between cyclophosphamide and bladder cancer, as well as between cyclophosphamide, busulfan, melphalan, and hematologic cancer. However, the non-significant association between cyclosporine and hematologic cancer implies that there may be insufficient evidence for cyclosporine to be categorized as a group I pharmaceutical. In conclusion, the results of this study are expected to enhance the persistent surveillance of group I pharmaceutical use, assist in establishing novel clinical strategies for patients with various indications, and provide additional evidence for re-categorizing current group I pharmaceuticals into other groups.

### Supplementary Information

Supplementary Information.

## Data Availability

All data generated or analyzed during this study are included in this published article and its supplementary information files.
