# Supplementary material for: Group I pharmaceuticals of IARC and associated cancer risks: systematic review and meta-analysis
Source: Sci Rep. 2024 Jan 3;14:413. doi: 10.1038/s41598-023-50602-6 (PMC10764325; doi:10.1038/s41598-023-50602-6)
Supplement: Supplementary file 1 — Supplementary Information. [file 41598_2023_50602_MOESM1_ESM.docx]

Original Research

**Group I pharmaceuticals of IARC and associated cancer risks: Systematic review and meta-analysis**

Woojin Lim^1,2,3^, Sungji Moon^1,2,4^, Na Rae Lee^5^, Ho Gyun Shin^5^, Su-Yeon Yu^5^, Jung Eun Lee^6^, Inah Kim^7^, Kwang-Pil Ko^8^, Sue K. Park^1,2,9^

^1^Department of Preventive Medicine, Seoul National University College of Medicine, Seoul 03080, Republic of Korea

^2^Cancer Research Institute, Seoul National University, Seoul 03080, Republic of Korea

^3^Department of Biomedical Sciences, Seoul National University Graduate School, Seoul 03080, Republic of Korea

^4^Interdisciplinary Program in Cancer Biology, Seoul National University College of Medicine, Seoul 03080, Republic of Korea

^5^National Evidence-based Healthcare Collaborating Agency (NECA), Seoul 04933, Republic of Korea

^6^Department of Food and Nutrition, Seoul National University College of Human Ecology, Seoul 08826, Republic of Korea

^7^Department of Occupational and Environmental Medicine, Hanyang University College of Medicine, Seoul 04763, Republic of Korea

^8^Clinical Preventive Medicine Center, Seoul National University Bundang Hospital, **Seongnam-si 13620, Gyeonggi-do,** Republic of Korea

^9^Integrated Major in Innovative Medical Science, Seoul National University College of Medicine, Seoul 03080, Republic of Korea

**Correspondence to:** Dr. Sue K. Park, MD, MPH, PhD.

Department of Preventive Medicine, Seoul National University College of Medicine, 103 Daehak-ro, Jongno-gu, Seoul, 03080, Korea.

Tel: +82-2-740-8338; E-mail: [suepark@snu.ac.kr](mailto:suepark@snu.ac.kr)

**Short title:** **Cancer risk associated with Group I pharmaceuticals**

**Supplementary Table list**

Supplementary Table 1. PICOTS-SD criteria of the systematic review.

Supplementary Table 2. Indication and outcome cancer sites of group I pharmaceuticals.

Supplementary Table 3. Search terms defined for the systematic review.

Supplementary Table 4. Studies on the association between cyclosporine treatment and subsequent cancer risk.

Supplementary Table 5. Studies on the association between azathioprine treatment and subsequent cancer risk.

Supplementary Table 6. Studies on the association between cyclophosphamide treatment and subsequent cancer risk.

Supplementary Table 7. Studies on the association between busulfan, chlorambucil, melphalan, methoxsalen + UV, thiotepa, etoposide, or MOPP on subsequent cancer risk.

Supplementary Table 8. PRISMA 2020 checklist of the systematic review.

**Supplementary Table 9. Sensitivity analysis: Influential meta-analysis of group-I pharmaceuticals and associated cancers.**

Supplementary Figure legends

Supplementary Figure 1. PRISMA flowchart of systematic reviews on cyclosporine and skin cancer.

Supplementary Figure 2. PRISMA flowchart of systematic reviews on cyclosporine and hematologic cancer.

Supplementary Figure 3. PRISMA flowchart of systematic reviews on azathioprine and skin cancer.

Supplementary Figure 4. PRISMA flowchart of systematic reviews on azathioprine and hematologic cancer.

Supplementary Figure 5. PRISMA flowchart of systematic reviews on cyclophosphamide and bladder cancer.

Supplementary Figure 6. PRISMA flowchart of systematic reviews on cyclophosphamide and hematologic cancer.

Supplementary Figure 7. PRISMA flowchart of systematic reviews on busulfan and hematologic cancer.

Supplementary Figure 8. PRISMA flowchart of systematic reviews on methoxsalen + UV and skin cancer.

Supplementary Figure 9. PRISMA flowchart of systematic reviews on melphalan and hematologic cancer.

Supplementary Figure 10. PRISMA flowchart of systematic reviews on chlorambucil and hematologic cancer.

Supplementary Figure 11. PRISMA flowchart of systematic reviews on thiotepa and hematologic cancer.

Supplementary Figure 12. PRISMA flowchart of systematic reviews on treosulfan and hematologic cancer.

Supplementary Figure 13. PRISMA flowchart of systematic reviews on MOPP and lung cancer.

Supplementary Figure 14. PRISMA flowchart of systematic reviews on MOPP and hematologic cancer.

Supplementary Figure 15. PRISMA flowchart of systematic reviews on BEP and hematologic cancer.

Supplementary Figure 16. PRISMA flowchart of systematic reviews on etoposide and hematologic cancer.

Supplementary Figure 17. Summarized results of the quality assessment for included studies using RoB and RoBANS.

Supplementary Figure 18. Individual results of the quality assessment for included literature using RoBANS.Supplementary Figure 19. Sensitivity analysis: Influence on overall SRR and contribution to overall heterogeneity of studies on association between cyclosporine and skin cancer.

Supplementary Figure 20. Sensitivity analysis: Influence on overall SRR and contribution to overall heterogeneity of studies on association between cyclosporine and hematologic cancer.

Supplementary Figure 21. Sensitivity analysis: Influence on overall SRR and contribution to overall heterogeneity of studies on association between azathioprine and skin cancer.

Supplementary Figure 22. Sensitivity analysis: Influence on overall SRR and contribution to overall heterogeneity of studies on association between azathioprine and hematologic cancer.

Supplementary Figure 23. Sensitivity analysis: Influence on overall SRR and contribution to overall heterogeneity of studies on association between cyclophosphamide and bladder cancer.

Supplementary Figure 24. Sensitivity analysis: Influence on overall SRR and contribution to overall heterogeneity of studies on association between cyclophosphamide and hematologic cancer.

Supplementary Figure 25. Sensitivity analysis: Influence on overall SRR and contribution to overall heterogeneity of studies on association between melphalan and hematologic cancer.

Supplementary Table 1. PICOTS-SD criteria of the systematic review.

| PICO-TSD | Definition |
| --- | --- |
| P (Population) | Patients with indication of group-I pharmaceuticals  (Indications with sufficient evidence from IARC monographs) |
| I (Intervention) | Exposure (ever use) of group-I pharmaceuticals |
| C (Comparison) | Population with non-exposure (never use) of group-I pharmaceuticals |
| O (Outcome) | Subsequent cancers with sufficient evidence from IARC monographs |
| T (Time) | Study period between 1990/1/1 and 2021/12/31 |
| S (Setting) | All epidemiological research settings included |
| SD (Study design) | Non-randomized studies including cohort and case-control (PCCS, HCCS, NCCS) studies and RCT |

Abbreviations: IARC, International Agency for Research on Cancer; PCCS, Population based case-control study; HCCS,

Hospital based case-control study; NCCS, Nested case-control study; RCT, randomized controlled trials.

Supplementary Table 2. Indication and outcome cancer sites of group I pharmaceuticals.

| **Group-I pharmaceuticals** | **Indication^1^** | | | | **Cancer site**  **(ICD-10)** |
| --- | --- | --- | --- | --- | --- |
| **Cyclosporine** | Graft-versus-host disease | Chronic allograft rejection | Solid organ transplantation | Rheumatoid arthritis | Skin(C43-C44), Hematologic(C81-C96) |
|  | Psoriasis | Atopic dermatitis | Keratoconjunctivitis sicca |  |  |
| **Azathioprine** | Solid organ transplantation | Rheumatoid arthritis | Inflammatory bowel disease | Myasthenia | Skin(C43-C44), Hematologic(C81-C96) |
|  | Acute lymphocytic leukemia | Polyangiitis | Systemic lupus erythematosus | Granulomatosis |  |
| **Cyclophosphamide** | Hodgkin lymphoma | Non-Hodgkin lymphoma | Multiple myeloma | Chronic/Acute lymphocytic leukemia | Urinary bladder (C67), Hematologic(C81-C96) |
|  | Chronic/Acute myeloid leukemia | Cutaneous T-cell lymphoma | Neuroblastoma | Ovarian cancer |  |
|  | Retinoblastoma | Breast cancer | Rheumatoid arthritis | Small cell cancer of the lung |  |
|  | Sarcoma | Nephrotic syndrome | Transplantation (Solid organ and bone marrow stem cell) | Polyangiitis |  |
|  | Granulomatosis |  |  |  |  |
| **Busulfan** | Chronic myeloid leukemia | Polycythemia Vera | Essential thrombocythemia | Stem cell transplantation | Hematologic(C81-C96) |
|  | Myelofibrosis |  |  |  |  |
| **Melphalan** | Multiple myeloma | Ovarian adenocarcinoma | Breast cancer | Neuroblastoma | Hematologic(C81-C96) |
|  | Polycythemia Vera | Malignant melanoma | Soft-tissue sarcoma | Amyloidosis |  |
|  | Bone marrow stem cell transplantation |  |  |  |  |
| **Chlorambucil** | Chronic lymphocytic leukemia | Waldenström macroglobulinemia | Indolent non-Hodgkin lymphoma | Polycythemia Vera | Hematologic(C81-C96) |
|  | Nephrotic syndrome | Ovarian cancer | Hodgkin lymphoma |  |  |
| **Methoxsalen + UV** | Psoriasis | Cutaneous T-cell lymphoma | Idiopathic vitiligo | Graft-versus-host disease | Skin(C43-C44) |
|  | Solid organ transplantation | eczema |  |  |  |
| **Thiotepa** | Urinary bladder cancer | Ovarian adenocarcinoma | Breast cancer | Thyroid cancer | Hematologic(C81-C96) |
| **Treosulfan** | Ovarian cancer | Stem cell transplantation | Hematologic malignancies | Solid tumors | Hematologic(C81-C96) |
| **Etoposide** | Tumors of the testis | Small cell cancer of the lung | Acute leukemia |  | Hematologic(C81-C96) |
| **BEP** | Tumors of the testis | Small cell cancer of the lung | Acute leukemia |  | Hematologic(C81-C96) |
| **MOPP** | Hodgkin lymphoma |  |  |  | Hematologic(C81-C96)  Lung (C34) |

Abbreviations: UV, ultraviolet; MOPP, Mustargen + Oncovin + Procarbazine + Prednisone; BEP, etoposide + bleomycin + cisplatin.

1. Above indications were diseases with sufficient evidence from the IARC monographs [1-4, 7, 10, 11]

Supplementary Table 3. Search terms defined for the systematic review.

| **Group-I pharmaceuticals** | **Outcome** | **MeSH Term** |
| --- | --- | --- |
| **Cyclosporine** | Skin cancer | ((((("Skin neoplasms"[MeSH Terms]) OR "Skin neoplasms"[All Fields]) OR "Skin cancer"[All Fields]) AND “Cyclosporine "[MeSH Terms]) AND " Cyclosporine "[All Fields]) AND ("1990/01/01"[Date - Publication] : "2021/12/31"[Date - Publication]) |
|  | Hematologic cancer | (((((("lymphoma"[MeSH Terms]) OR "lymphoma"[All fields]) OR "leukemia"[MeSH Terms]) OR "leukemia"[All fields]) AND "Cyclosporine"[MeSH Terms]) AND " Cyclosporine "[All fields]) AND ("1990/01/01"[Date - Publication] : "2021/12/31"[Date - Publication]) |
| **Azathioprine** | Skin cancer | ((((("Skin neoplasms"[MeSH Terms]) OR "Skin neoplasms"[All Fields]) OR "Skin cancer"[All Fields]) AND “Azathioprine "[MeSH Terms]) AND " Azathioprine "[All Fields]) AND ("1990/01/01"[Date - Publication] : "2021/12/31"[Date - Publication]) |
|  | Hematologic cancer | (((((("lymphoma"[MeSH Terms]) OR "lymphoma"[All fields]) OR "leukemia"[MeSH Terms]) OR "leukemia"[All fields]) AND " Azathioprine "[MeSH Terms]) AND " Azathioprine "[All fields]) AND ("1990/01/01"[Date - Publication] : "2021/12/31"[Date - Publication]) |
| **Cyclophosphamide** | Bladder cancer | ((((("Bladder neoplasms"[MeSH Terms]) OR "Bladder neoplasms"[All Fields]) OR "Bladder cancer"[All Fields]) AND “Cyclophosphamide "[MeSH Terms]) AND " Cyclophosphamide "[All Fields]) AND ("1990/01/01"[Date - Publication] : "2021/12/31"[Date - Publication]) |
|  | Hematologic cancer | (((((("lymphoma"[MeSH Terms]) OR "lymphoma"[All fields]) OR "leukemia"[MeSH Terms]) OR "leukemia"[All fields]) AND " Cyclophosphamide "[MeSH Terms]) AND " Cyclophosphamide "[All fields]) AND ("1990/01/01"[Date - Publication] : "2021/12/31"[Date - Publication]) |
| **Busulfan** | Hematologic cancer | (((((("lymphoma"[MeSH Terms]) OR "lymphoma"[All fields]) OR "leukemia"[MeSH Terms]) OR "leukemia"[All fields]) AND " Busulfan "[MeSH Terms]) AND " Busulfan "[All fields]) AND ("1990/01/01"[Date - Publication] : "2021/12/31"[Date - Publication]) |
| **Methoxsalen + UV** | Skin cancer | ((((("Skin neoplasms"[MeSH Terms]) OR "Skin neoplasms"[All Fields]) OR "Skin cancer"[All Fields]) AND “Methoxsalen "[MeSH Terms]) AND " Methoxsalen "[All Fields]) AND ("1990/01/01"[Date - Publication] : "2021/12/31"[Date - Publication]) |
| **Melphalan** | Hematologic cancer | (((((("lymphoma"[MeSH Terms]) OR "lymphoma"[All fields]) OR "leukemia"[MeSH Terms]) OR "leukemia"[All fields]) AND " Melphalan "[MeSH Terms]) AND " Melphalan "[All fields]) AND ("1990/01/01"[Date - Publication] : "2021/12/31"[Date - Publication]) |
| **Chlorambucil** | Hematologic cancer | (((((("lymphoma"[MeSH Terms]) OR "lymphoma"[All fields]) OR "leukemia"[MeSH Terms]) OR "leukemia"[All fields]) AND " Chlorambucil "[MeSH Terms]) AND " Chlorambucil "[All fields]) AND ("1990/01/01"[Date - Publication] : "2021/12/31"[Date - Publication]) |
| **Thiotepa** | Hematologic cancer | (((((("lymphoma"[MeSH Terms]) OR "lymphoma"[All fields]) OR "leukemia"[MeSH Terms]) OR "leukemia"[All fields]) AND " Thiotepa "[MeSH Terms]) AND " Thiotepa "[All fields]) AND ("1990/01/01"[Date - Publication] : "2021/12/31"[Date - Publication]) |
| **Treosulfan** | Hematologic cancer | (((((("lymphoma"[MeSH Terms]) OR "lymphoma"[All fields]) OR "leukemia"[MeSH Terms]) OR "leukemia"[All fields]) AND " Treosulfan "[MeSH Terms]) AND " Treosulfan "[All fields]) AND ("1990/01/01"[Date - Publication] : "2021/12/31"[Date - Publication]) |
| **MOPP** | Lung cancer | ((((("Lung neoplasms"[MeSH Terms]) OR "Lung neoplasms"[All Fields]) OR "Lung cancer"[All Fields]) AND “MOPP "[MeSH Terms]) AND " MOPP "[All Fields]) AND ("1990/01/01"[Date - Publication] : "2021/12/31"[Date - Publication]) |
|  | Hematologic cancer | (((((("lymphoma"[MeSH Terms]) OR "lymphoma"[All fields]) OR "leukemia"[MeSH Terms]) OR "leukemia"[All fields]) AND " MOPP "[MeSH Terms]) AND " MOPP "[All fields]) AND ("1990/01/01"[Date - Publication] : "2021/12/31"[Date - Publication]) |
| **BEP** | Hematologic cancer | (((((((((("leukemia"[All fields]) OR "leukemia"[MeSH Terms]) OR "lymphoma"[All fields]) OR "lymphoma"[MeSH Terms]) AND "etoposide"[MeSH Terms]) AND "etoposide"[All fields]) AND "cisplatin"[MeSH Terms]) AND "cisplatin"[All fields]) AND "bleomycin"[MeSH Terms]) AND "bleomycin"[All fields]) AND ("1990/01/01"[Date - Publication] : "2021/12/31"[Date - Publication]) |
| **Etoposide** | Hematologic cancer | (((((("lymphoma"[MeSH Terms]) OR "lymphoma"[All fields]) OR "leukemia"[MeSH Terms]) OR "leukemia"[All fields]) AND " Etoposide "[MeSH Terms]) AND " Etoposide "[All fields]) AND ("1990/01/01"[Date - Publication] : "2021/12/31"[Date - Publication]) |

Abbreviations: UV, ultraviolet; MOPP, Mustargen + Oncovin + Procarbazine + Prednisone; BEP, etoposide + bleomycin + cisplatin; MeSH, Medical Subject Headings.

Supplementary Table 4. Studies on the association between cyclosporine treatment and subsequent cancer risk.

| Outcome | Indications | Design | Author,  year | Study  region | Study period | Study subjects | | - Ex/Non-Ex   Ca/Co | RR (95% CI) | Adjustment |
| --- | --- | --- | --- | --- | --- | --- | --- | --- | --- | --- |
|  |  |  |  |  |  | **N** | **Age** |  |  |  |
| **Skin cancer** | Solid organ transplant | - Cohort | Clarke et al., 2015 | U.S | 1987-2009 | 189,498 | ≥20 | - 71/39^1^ | 1.50 (1.00-2.30) | Age, sex, time since transplant, and other maintenance regimens |
|  |  |  | Bhat et al.,  2018 | U.S | 1987-2015 | 534,472 | ≥18 | - 166,007/ - 368,465 | 1.14 (1.04-1.24) | NR |
|  |  |  | Mithoefer et al., 2002 | U.S | 1991-2000 | 151 | ≥18 | - 56/95 | 4.43 (1.68-11.60) | NR |
|  |  |  | Kasiske et al., 2004 | U.S | 1995-2001 | 35,765 | ≥0 | - 5,995/29,770 | 1.02 (0.86-1.21) | Age, race, education, occupation, living status, HBV/HCV serology, major histocompatibility mismatches, panel reactive antibodies |
|  |  |  | Jensen et al., 1999 | Norway | 1963-1992 | 2,076 | 0.8-82.5 | - 1,323/753 | 2.80 (1.40-5.30) | Age (transplantation and graft organ) |
|  |  |  | Keller et al., 2010 | Switzerland | 2002-2005 | 243 | 19-79 | - 208/35 | 2.03 (0.32-12.98) | NR |
|  |  |  | Hamandi et al., 2018 | U.S, Canada Europe Australia | 2005-2008 | 900 | ≥18 | - 518/382 | 0.64 (0.38-1.08) | Exposure to voriconazole, other azoles, and immunosuppressive agents (Time-varying covariates) |
|  |  |  | Molina et al., 2010 | Spain | 1984-2004 | 3,393 | >15 | - 2,864/529 | 1.10 (0.80-1.50) | Age, sex, smoking, and immunosuppression in the first 3 months with mycophenolate mofetil and/or tacrolimus |
|  |  |  | Cahoon et al., 2018 | U.S | 1987-2014 | 244,964 | ≥15 | - 74/89 | 0.81 (0.55-1.19) | Sex, race, US citizenship, age at transplant (years), calendar year of transplant, and time since transplant (years) |
|  |  | - NCCS | Coghill et al., 2016 | U.S | 1995-2012 | 2,004 | ≥18 | - 75/140 | 1.02 (0.64-1.63) | Matched for sex, age, race, transplant year, hospital, donor type, transplanted organ, and time between transplantation and interview. Adjusted for family history of skin cancer and personal history of precancerous lesions |
|  |  |  | Ingvar et al., 2010 | Sweden | 1970-1997 | 5,931 | 11-71 | - 159/149 | 1.80 (0.80-3.80) | Matched to age and calendar period between transplantation and cancer diagnosis.  Adjusted for recipient’s sex, accumulated dose of azathioprine, cyclosporine and corticosteroids over follow-up period |
|  | Rheumatoid arthritis | - Cohort | Lange et al., 2016 | Australia | 1978-2005 | - 375 | 60.0  (mean) | - 34/341 | 2.51 (1.23-5.13) | Age at commencement, treatment with methotrexate, and weeks of methotrexate use |
|  |  | - PCCS | Tseng et al., 2018 | Taiwan | 1995-2013 | - 127/53 | ≥18 | - 31/5 | 5.70 (2.20-14.86) | Matched for age, sex, and reference date.  Adjusted for residential regions, occupation, diabetes mellitus, coronary artery disease, hypertension, chronic obstructive pulmonary disease, chronic kidney disease, organ transplant |
| **Hematologic**  **cancer** |  |  |  |  |  |  |  |  |  |  |
| Hematologic  cancer | Solid organ transplant | Cohort | Bhat et al.,  2018 | U.S | 1987-2015 | 534,472 | ≥18 | - 166,007/ - 368,465 | 1.00 (0.84-1.20) | NR |
| NHL | Solid organ transplant | Cohort | Na et al.,  2016 | Australia | 1984-2006 | - 4,131 | 49  (median) | - 2,777/956^2^ | 0.73 (0.37-1.47) | Age at transplantation, sex, transplant year, transplanted organ plus muromonab-CD3 induction and other immunosuppressive agents in the respective category |
|  |  | - NCCS | Fernberg et al., 2011 | Sweden | 1970-2008 | - 11,081 | 0-87 | - 31/84 | 0.90 (0.20-5.80) | Matched to the cases by age and calendar period of transplantation; or Adjusted for the matching factors |
| LPD | Solid organ transplant | - Cohort | Caillard et al., 2006 | U.S | 1991-2000 | - 66,159 | ≥20 | - NR | 0.80 (0.61-1.05) | Recipient’s age, sex, and race, cause of ESRD, duration of dialysis, donor type, donor age, CMV, HCV, HBV, and HIV status, HLA match level, acute rejection during the first year after transplantation, induction or rejection therapy and for treatment by cyclosporine, tacrolimus, mycophenolate, azathioprine, sirolimus and steroids at discharge and maintenance therapy |
|  |  |  | O’ Regan et al., 2017 | Ireland | 1991-2010 | - 1,996 | ≥18 | - 139/1,857 | 1.03 (0.87-1.22) | NR |
|  |  |  | Caillard et al., 2012 | France | 1998-2007 | - 20,728 | ≥18 | - 10,656/ - 10,072 | 0.66 (0.36-1.19) | NR |

Abbreviations: N, number; RR, relative risk; CI, confidence interval; Ex/Non-Ex, N of exposed group / N of non-exposed group; Ca/Co, N of Cases/N of Controls; NCCS, Nested case-control study; PCCS, Population based case-control study; ESRD, End-stage renal disease; ; CMV, Cytomegalovirus; HCV, Hepatitis C virus; HBV, Hepatitis B virus; HIV, Human immunodeficiency virus; HLA, [Human Leukocyte Antigen;](https://bethematch.org/transplant-basics/matching-patients-with-donors/how-donors-and-patients-are-matched/hla-basics/) NR, Not reported; LPD, Lymphoproliferative disorder; NHL, Non-Hodgkin lymphoma; MDS, myelodysplastic syndrome. 1. Exposed/Non-exposed number of outcome patients. 2. Exposed/Non-exposed number do not add up to the total number due to missing data. Hematologic cancer includes all types of lymphoma and leukemia.

Supplementary Table 5. Studies on the association between azathioprine treatment and subsequent cancer risk.

| Outcome | Indications | Design | Author,  year | Study  region | Study period | Study subjects | | - Ex/Non-Ex   Ca/Co | RR (95% CI) | Adjustment |
| --- | --- | --- | --- | --- | --- | --- | --- | --- | --- | --- |
|  |  |  |  |  |  | **N** | **Age** |  |  |  |
| **Skin cancer** | Solid organ transplant | - Cohort | Clarke et al., 2015 | U.S | 1987-2009 | 189,498 | ≥20 | 44/66^1^ | 1.50 (1.00-2.10) | Age, sex, time since transplant, and other maintenance regimens |
|  |  |  | Kasiske et al., 2004 | U.S | 1995-2001 | - 35,765 | - ≥0 | - 7,231/28,534 | 1.17 (1.01-1.37) | Age, race, education, occupation, living status, HBV/HCV serology, major histocompatibility mismatches, panel reactive antibodies |
|  |  |  | Hamandi et al., 2018 | U.S, Canada Europe Australia | 2005-2008 | 900 | ≥18 | - 337/563 | 1.21 (0.71-2.07) | Exposure to voriconazole, other azoles, and immunosuppressive agents (Time-varying covariates) |
|  |  |  | Molina et al., 2010 | Spain | 1984-2004 | 3,393 | >15 | - 2,334/1,059 | 1.50 (1.10-1.90) | Age, sex, smoking, and immunosuppression in the first 3 months with mycophenolate mofetil and/or tacrolimus |
|  |  |  | Keller et al., 2010 | Switzerland | 2002-2005 | 243 | 19-79 | - 106/137 | 1.82 (0.51-6.56) | NR |
|  |  |  | Cahoon et al., 2018 | U.S | 1987-2014 | 244,964 | ≥15 | - 42/121 | 0.86 (0.55-1.31) | Sex, race, US citizenship, age at transplant (years), calendar year of transplant, and time since transplant (years) |
|  |  | - NCCS | Coghill et al.,  2016 | U.S | 1995-2012 | 2,004 | ≥18 | - 19/18 | 2.67 (1.23-5.76) | Matched for sex, age, race, transplant year, hospital, donor type, transplanted organ, and time between transplantation and interview. Adjusted for family history of skin cancer and personal history of precancerous lesions |
|  |  |  | Ingvar et al., 2010 | Sweden | 1970-1997 | 5,931 | 11-71 | - 198/160 | 6.00 (2.10-17.50) | Matched to age and calendar period between transplantation and cancer diagnosis.  Adjusted for recipient’s sex, accumulated dose of azathioprine, cyclosporine and corticosteroids over follow-up period |
|  | Inflammatory bowel disease | Cohort | Pasternak et al., 2013 | Denmark | 1997-2008 | - 45,986 | 38 (mean) | - 11/162^1^ | 1.67 (0.86-3.21) | Baseline propensity scores and the following time-varying covariates: age, calendar year, disease duration, inflammatory bowel disease hospitalizations in the last year and use of aminosalicylates, oral corticosteroids, enteral or rectal corticosteroids, and other immunosuppressants |
|  |  |  | Setshedi et al., 2012 | South Africa | 1960-2007 | - 1,084 | 34  (median) | - 123/836^2^ | 5.10 (1.10-22.80) | NR |
|  |  | - NCCS | Singh et al., 2011 | Canada | 1995-2009 | - 100,996 | 36  (median) | - 29/1,522 | 1.31 (0.85-2.03) | Matched for age and sex.  Adjusted for socioeconomic status and health care use |
|  | Myasthenia | - PCCS | Pedersen et al., 2014 | Denmark | 1995-2009 | - 30/360 | 70  (median) | - 16/107 | 3.30 (1.50-7.30) | Matched for age and sex.  Adjusted for duration of myasthenia and Charlson Comorbidity Index |
|  | Rheumatoid arthritis + Psoriasis | - Cohort | Lange et al., 2016 | Australia | 1978-2005 | - 375 | 60.0  (mean) | - 21/354 | 1.94 (0.87-4.34) | Age at commencement, treatment with methotrexate, and weeks of methotrexate use |
| **Hematologic cancer** |  |  |  |  |  |  |  |  |  |  |
| Burkitt lymphoma | Solid organ transplant | Cohort | Mbulaiteye  et al., 2013 | U.S | 1987-2009 | - 203,557 | 47  (median) | - 25/74^1^ | 0.56 (0.34-0.89) | Age at transplant, sex, race, transplanted organ, and EBV |
| NHL | Solid organ transplant | - NCCS | Fernberg et al., 2011 | Sweden | 1970-2008 | - 11,081 | 0-87 | - 34/77 | 2.80 (0.80-16.00) | Matched for age and calendar period of transplantation; Or Adjusted for the matching factors |
| LPD | Solid organ transplant | - Cohort | Caillard et al., 2006 | U.S | 1991-2000 | - 66,159 | ≥20 | - NR | 0.87 (0.67-1.14) | Recipient’s age, sex, and race, cause of ESRD, duration of dialysis, donor type, donor age, CMV, HCV, HBV, and HIV status, HLA match level, acute rejection during the first year after transplantation, induction or rejection therapy and for treatment by cyclosporine, tacrolimus, mycophenolate, azathioprine, sirolimus and steroids at discharge and maintenance therapy |
|  |  | Cohort | O’ Regan et al., 2017 | Ireland | 1991-2010 | - 1,996 | ≥18 | - 135/1,861 | 0.95 (0.85-1.07) | NR |
|  |  | Cohort | Caillard et al., 2012 | France | 1998-2007 | - 20,640 | ≥18 | - 2,274/ - 18,366 | 1.30 (0.76-2.19) | NR |
|  |  | Cohort | Zimmerman  et al., 2010 | Germany | 1998-2008 | - 431 | 16.1-73.6 | - 71/355 | 4.34 (1.23-15.75) | NR |
|  |  | Cohort | Na et al.,  2016 | Australia | 1984-2006 | - 4,131 | 49  (median) | - 2,316/1382^2^ | 1.88 (1.03-3.41) | Age at transplantation, sex, transplant year, transplanted organ plus muromonab-CD3 induction and other immunosuppressive agents in the respective category |
| Hematologic cancer | Rheumatoid arthritis | - NCCS | Bernatsky et al.,  2008 | Canada | 1980-2003 | - 23,810 | 61.7  (mean) | - 40/285 | 1.07 (0.74-1.54) | Matched for age and sex.  Adjusted for all concomitant drug exposures, number of physician visits up to index date, and extra-articular disease |
| Hematologic cancer | systemic lupus erythematosus | - Cohort | Bernatsky et al., 2008 | Canada | 1958-2000 | - 784 | 35/42  (mean) | - 246/538 | 1.19(0.48-2.92) | Anti-malarial agents, Systemic glucocorticoids, NSAIDs, Aspirin, Tobacco use, Age >65, sex, White, Damage, Residence in North America, Sjogren syndrome, Cohort entry before 1990 |
| Lymphoma | Inflammatory bowel disease | Cohort | Pasternak et al., 2013 | Denmark | 1997-2008 | - 45,986 | 38  (mean) | - 9/80^1^ | 2.40 (1.13-5.11) | Baseline propensity scores and the following time-varying covariates: age, calendar year, disease duration, inflammatory bowel disease hospitalizations in the last year and use of aminosalicylates, oral corticosteroids, enteral or rectal corticosteroids, and other immunosuppressants |
|  |  |  | Khan et al.,  2013 | U.S | 2001-2011 | - 36,891 | 60 (median) | - 3,126/33,765 | 4.20 (2.50-6.80) | Age, sex, and race |
| LPD | Inflammatory bowel disease | Cohort | Beaugerie et al., 2009 | France | 2004-2007 | - 19,486 | 40.3  (mean) | - 5,867/13,619 | 5.28 (2.01-13.90) | Age, sex, thiopurine therapy status |

Abbreviations: N, number; RR, relative risk; CI, confidence interval; Ex/Non-Ex, N of exposed group / N of non-exposed group; Ca/Co, N of Cases/N of Controls; NCCS, Nested case-control study; PCCS, Population based case-control study; ESRD, End-stage renal disease; ; CMV, Cytomegalovirus; HCV, Hepatitis C virus; HBV, Hepatitis B virus; HIV, Human immunodeficiency virus; HLA, [Human Leukocyte Antigen;](https://bethematch.org/transplant-basics/matching-patients-with-donors/how-donors-and-patients-are-matched/hla-basics/) NR, Not reported; LPD, Lymphoproliferative disorder; NHL, Non-Hodgkin lymphoma. 1. Exposed/Non-exposed number of outcome patients. 2. Exposed/Non-exposed number do not add up to the total number due to missing data. Hematologic cancer includes all types of lymphoma and leukemia.

Supplementary Table 6. Studies on the association between cyclophosphamide treatment and subsequent cancer risk.

| Outcome | Indications | Design | Author,  year | Study  region | Study period | Study subjects | | - Ex/Non-Ex   Ca/Co | RR (95% CI) | Adjustment |
| --- | --- | --- | --- | --- | --- | --- | --- | --- | --- | --- |
|  |  |  |  |  |  | **N** | **Age** |  |  |  |
| **Bladder cancer** | Ovarian cancer | NCCS | Kaldor  et al., 1995 | Canada Denmark Finland Norway Germany Slovenia U.K Hospitals | 1960-1987 | 90,090 | 60 (mean) | 11/20 | 4.20 (1.20-14.00) | Matched for age, year of ovarian cancer diagnosis and survival time. |
|  | NHL | NCCS | Travis et al., 1995 | Netherlands U.S, Canada Sweden | 1965-1980 | 6,171 | 58 (median) | 9/20 | 4.50 (1.50-13.60) | Matched for cancer registry, sex, age, race, calendar year of diagnosis of NHL, and length of follow-up at least as long as the interval between the case subject's diagnosis of NHL and secondary cancer |
|  |  |  | Xu et al., 2013 | China | 1990-2011 | 3,412 | ≥0 | 9/566 | 1.09 (0.30-3.98) | Matched for sex, race, age at NHL diagnosis, calendar year of diagnosis of NHL, and length of follow-up at least as long as the interval of SMN development  Adjusted for radiation dose and field size, and Autologous stem-cell transplant and splenectomy |
| **Hematologic cancer** |  |  |  |  |  |  |  |  |  |  |
| Acute nonlymphocytic leukemia | Breast cancer | - NCCS | Curtis et al., 1992 | U.S | 1973-1985 | 82,700 | ≥0 | 14/32 | 3.10 (1.30-7.70) | Matched for registry, age and calendar year at breast-cancer diagnosis, race or ethnic group, and latency.  Adjusted for radiotherapy |
| Hematologic cancer | Rheumatoid arthritis | - NCCS | Bernatsky  et al., 2008 | Canada | 1980-2003 | - 23,810 | 61.7 (mean) | - 36/167 | 1.84 (1.24-2.73) | Matched for age and sex. Adjusted for all concomitant drug exposures, number of physician visits up to index date, and extra-articular disease |
| Hematologic cancer | systemic lupus erythematosus | Cohort | Bernatsky  et al., 2008 | Canada | 1958-2000 | 784 | 35/42  (mean) | 246/538 | 2.09 (0.69-6.30) | Anti-malarial agents, Systemic glucocorticoids, NSAIDs, Aspirin, Tobacco use, Age >65, sex, White, Damage, Residence in North America, Sjogren syndrome, Cohort entry before 1990 |
| Leukemia | Ovarian cancer | NCCS | Kaldor et al., 1990 | U.K | 1970-1985 | 99,113 | 58 (mean) | 19/38 | 2.65 (1.64-4.26) | NR |
|  | Lymphoma | PCCS | Nandakumar et al.,1991 | U.S | 1974-1986 | 97/194 | 22-96 | 20/13 | 14.80 (3.70-59.40) | Matched for age, date of diagnosis, and site of initial primary cancer among residents of 13 counties in western Washington State |
|  | NHL | NCCS | Travis et al., 1994 | Netherlands Sweden, U.S, Canada | 1965-1989 | 11,386 | 2.4-18.2 | 11/43 | 1.80 (0.70-4.90) | Matched for cancer registry, sex, race (Iowa only), age at NHL diagnosis, calendar year of diagnosis of Non-Hodgkin lymphoma, and length of follow-up |
|  |  | NCCS | Xu et al., 2013 | China | 1990-2011 | 3,412 | ≥0 | 32/543 | 1.20 (0.28-5.03) | Matched for sex, race, age at NHL diagnosis, calendar year of diagnosis of NHL, and length of follow-up at least as long as the interval of SMN development. Adjusted for radiation dose and field size, and autologous stem-cell transplant and splenectomy |

Abbreviations: N, number; RR, relative risk; CI, confidence interval; Ex/Non-Ex, N of exposed group / N of non-exposed group; Ca/Co, N of Cases/N of Controls; NCCS, Nested case-control study; PCCS, population-based case control study; HCCS, hospital-based case-control study; NHL, Non-Hodgkin lymphoma; SMN, second malignant neoplasm; NR, Not reported; RCT, randomized controlled trial.

Supplementary Table 7. Studies on the association between busulfan, chlorambucil, melphalan, methoxsalen + UV, thiotepa, etoposide, or MOPP on subsequent cancer risk.

| Group-I pharmaceuticals | Outcome | Indications | Design | Author,  year | Study  region | Study period | Study subjects | | Ex/Non-Ex Ca/Co | RR (95% CI) | Adjustment |
| --- | --- | --- | --- | --- | --- | --- | --- | --- | --- | --- | --- |
|  |  |  |  |  |  |  | **N** | **Age** |  |  |  |
| **Busulfan** | Hematologic cancer (Leukemia) | Essential thrombocythemia | - RCT | Finazzi et al., 2000 | Italy | 1990-1993 | 114 | 67 (median) | 15/99 | 4.48 (1.11-27.10) | NR |
|  | AML/MDS | Polycythemia vera | Cohort | Finazzi et al., 2005 | Italy | 1964-2001 | 1,638 | 62.1 | 61/1,577 | 8.64 (2.44-30.60) | Sex, age, disease duration, treatment at recruitment, presence of splenomegaly, cholesterol level  (2 categories), and hematologic parameters at baseline (2 categories) |
| **Chlorambucil** | Hematologic cancer (Acute nonlymphocytic leukemia) | NHL | NCCS | Travis et al., 1994 | Netherlands Sweden, U.S, Canada | 1965-1989 | 11,386 | 2.4-18.2 | 6/22 | 2.40 (0.70-8.60) | Matched for cancer registry, sex, race, age at NHL diagnosis, calendar year of diagnosis of NHL, and length of follow-up |
|  | Leukemia | Ovarian cancer | NCCS | Kaldor et al., 1990 | U.K | 1970-1985 | 99,113 | 58 (mean) | 11/10 | 1.19 (0.72-1.97) | NR |
| **Melphalan** | Hematologic cancer (Acute nonlymphocytic leukemia) | Breast cancer | NCCS | Curtis et al., 1992 | U.S | 1973-1985 | 82,700 | ≥0 | 14/32 | 3.10 (1.30-7.70) | Matched for registry, age and calendar year at breast cancer diagnosis, race or ethnic group, and latency. Adjusted for radiotherapy |
|  | Leukemia | Ovarian cancer | NCCS | Kaldor et al., 1990 | U.K | 1970-1985 | 99,113 | 58 (mean) | 32/42 | 1.88 (1.33-2.65) | NR |
|  | Leukemia | Ovarian cancer | NCCS | Travis et al., 1999 | U.S,  Europe | 1980-1993 | 28,971 | ≥0 | 28/40 | 20.8 (6.3–68.3) | Matched for registry, age when ovarian cancer was diagnosed, year of diagnosis of ovarian cancer, and survival without a second primary cancer for at least as long as the interval between the case patient’s diagnoses of ovarian cancer and leukemia |
| **Methoxsalen + UV** | Skin cancer | Psoriasis | NCCS | Hannuksela  et al., 2000 | Finland | 1973-1995 | 5,687 | ≥0 | 12/14 | 6.50 (1.40-31.40) | Matched for age, sex, and duration of psoriasis. Adjusted for other treatments, sex, year of birth, and the period between the onset of psoriasis |
| **Thiotepa** | Leukemia | Ovarian cancer | NCCS | Kaldor et al., 1990 | U.K | 1970-1985 | 99,113 | 58 (mean) | 11/18 | 1.82 (1.09-3.03) | NR |
| **MOPP** | Lung cancer | HD | - NCCS | Travis et al., 2002 | U.S, Canada Denmark Finland Sweden Netherlands | 1965-1994 | 19,046 | 9-81 | 55/92 | 5.00 (2.10-13.60) | Matched for registry, sex, calendar year, age at diagnosis of HD, and survival without a second cancer at least as long as the period from HD to lung cancer in the case patient. Adjusted for radiation dose and smoking status |
| **Etoposide** | Leukemia | Solid tumor | HCCS | Le Deley et al., 2003 | France | 1980-1999 | 257 | 7.8/7.8 (median) | 61/196 | 2.70 (1.20-6.00) | matched for sex, age at initial diagnosis, date of diagnosis, and hospital (when possible) |

Abbreviations: N, number; RR, relative risk; CI, confidence interval; Ex/Non-Ex, N of exposed group / N of non-exposed group; Ca/Co, N of Cases/N of Controls; UV, ultraviolet; MOPP, Mustargen-oncovin-procarbazine-prednisone mixture; ABVD, doxorubicin, bleomycin, vinblastine and dacarbazine; NCCS, nested case-control study; HCCS, hospital-based case control study; NR, Not reported; NHL, Non-Hodgkin lymphoma; HD, Hodgkin’s disease; MDS, myelodysplastic syndrome; AML, acute myeloid leukemia; BEP, etoposide + bleomycin + cisplatin; RCT, Randomized Controlled Trial.

Supplementary Table 8. PRISMA 2020 checklist of the systematic review.

| **Section and Topic** | **Item #** | **Checklist item** | **Location where item is reported** |
| --- | --- | --- | --- |
| **TITLE** | | |  |
| Title | 1 | Identify the report as a systematic review. | Page 1 |
| **ABSTRACT** | | |  |
| Abstract | 2 | See the PRISMA 2020 for Abstracts checklist. | N/A |
| **INTRODUCTION** | | |  |
| Rationale | 3 | Describe the rationale for the review in the context of existing knowledge. | Page 3 |
| Objectives | 4 | Provide an explicit statement of the objective(s) or question(s) the review addresses. | Page 3-4 |
| **METHODS** | | |  |
| Eligibility criteria | 5 | Specify the inclusion and exclusion criteria for the review and how studies were grouped for the syntheses. | Page 4-5 |
| Information sources | 6 | Specify all databases, registers, websites, organisations, reference lists and other sources searched or consulted to identify studies. Specify the date when each source was last searched or consulted. | Page 5-6 |
| Search strategy | 7 | Present the full search strategies for all databases, registers and websites, including any filters and limits used. | ST4 |
| Selection process | 8 | Specify the methods used to decide whether a study met the inclusion criteria of the review, including how many reviewers screened each record and each report retrieved, whether they worked independently, and if applicable, details of automation tools used in the process. | Page 6 |
| Data collection process | 9 | Specify the methods used to collect data from reports, including how many reviewers collected data from each report, whether they worked independently, any processes for obtaining or confirming data from study investigators, and if applicable, details of automation tools used in the process. | Page 6 |
| Data items | 10a | List and define all outcomes for which data were sought. Specify whether all results that were compatible with each outcome domain in each study were sought (e.g. for all measures, time points, analyses), and if not, the methods used to decide which results to collect. | Page 5 |
|  | 10b | List and define all other variables for which data were sought (e.g. participant and intervention characteristics, funding sources). Describe any assumptions made about any missing or unclear information. | ST2 |
| Study risk of bias assessment | 11 | Specify the methods used to assess risk of bias in the included studies, including details of the tool(s) used, how many reviewers assessed each study and whether they worked independently, and if applicable, details of automation tools used in the process. | Page 6 |
| Effect measures | 12 | Specify for each outcome the effect measure(s) (e.g. risk ratio, mean difference) used in the synthesis or presentation of results. | Page 5-6 |
| Synthesis methods | 13a | Describe the processes used to decide which studies were eligible for each synthesis (e.g. tabulating the study intervention characteristics and comparing against the planned groups for each synthesis (item #5)). | Page 5-6 |
|  | 13b | Describe any methods required to prepare the data for presentation or synthesis, such as handling of missing summary statistics, or data conversions. | N/A |
|  | 13c | Describe any methods used to tabulate or visually display results of individual studies and syntheses. | ST5-8 |
|  | 13d | Describe any methods used to synthesize results and provide a rationale for the choice(s). If meta-analysis was performed, describe the model(s), method(s) to identify the presence and extent of statistical heterogeneity, and software package(s) used. | Page 6 |
|  | 13e | Describe any methods used to explore possible causes of heterogeneity among study results (e.g. subgroup analysis, meta-regression). | Page 6 |
|  | 13f | Describe any sensitivity analyses conducted to assess robustness of the synthesized results. | Page 6 |
| Reporting bias assessment | 14 | Describe any methods used to assess risk of bias due to missing results in a synthesis (arising from reporting biases). | Page 6 |
| Certainty assessment | 15 | Describe any methods used to assess certainty (or confidence) in the body of evidence for an outcome. | N/A |
| **RESULTS** | | |  |
| Study selection | 16a | Describe the results of the search and selection process, from the number of records identified in the search to the number of studies included in the review, ideally using a flow diagram. | SF1-16 |
|  | 16b | Cite studies that might appear to meet the inclusion criteria, but which were excluded, and explain why they were excluded. | SF1-16 |
| Study characteristics | 17 | Cite each included study and present its characteristics. | N/A |
| Risk of bias in studies | 18 | Present assessments of risk of bias for each included study. | SF17-18 |
| Results of individual studies | 19 | For all outcomes, present, for each study: (a) summary statistics for each group (where appropriate) and (b) an effect estimate and its precision (e.g. confidence/credible interval), ideally using structured tables or plots. | T2-6 |
| Results of syntheses | 20a | For each synthesis, briefly summarise the characteristics and risk of bias among contributing studies. | Page 8 |
|  | 20b | Present results of all statistical syntheses conducted. If meta-analysis was done, present for each the summary estimate and its precision (e.g. confidence/credible interval) and measures of statistical heterogeneity. If comparing groups, describe the direction of the effect. | T2 |
|  | 20c | Present results of all investigations of possible causes of heterogeneity among study results. | T2-6 |
|  | 20d | Present results of all sensitivity analyses conducted to assess the robustness of the synthesized results. | T2-6 |
| Reporting biases | 21 | Present assessments of risk of bias due to missing results (arising from reporting biases) for each synthesis assessed. | Page 8 |
| Certainty of evidence | 22 | Present assessments of certainty (or confidence) in the body of evidence for each outcome assessed. | N/A |
| **DISCUSSION** | | |  |
| Discussion | 23a | Provide a general interpretation of the results in the context of other evidence. | Page 9-10 |
|  | 23b | Discuss any limitations of the evidence included in the review. | Page 12 |
|  | 23c | Discuss any limitations of the review processes used. | Page 12 |
|  | 23d | Discuss implications of the results for practice, policy, and future research. | Page 12 |
| **OTHER INFORMATION** | | |  |
| Registration and protocol | 24a | Provide registration information for the review, including register name and registration number, or state that the review was not registered. | N/A |
|  | 24b | Indicate where the review protocol can be accessed, or state that a protocol was not prepared. | N/A |
|  | 24c | Describe and explain any amendments to information provided at registration or in the protocol. | N/A |
| Support | 25 | Describe sources of financial or non-financial support for the review, and the role of the funders or sponsors in the review. | Page 13 |
| Competing interests | 26 | Declare any competing interests of review authors. | Page 13 |
| Availability of data, code and other materials | 27 | Report which of the following are publicly available and where they can be found: template data collection forms; data extracted from included studies; data used for all analyses; analytic code; any other materials used in the review. | Page 6 |

**Supplementary Table 9. Sensitivity analysis: Influential meta-analysis of group-I pharmaceuticals and associated cancers.**

| **Pharmaceuticals** | **Outcome** | **Omitting Studies** | **RR (95% CI)** | **p-value** | **Tau^2^** | **Tau** | **I^2^** |
| --- | --- | --- | --- | --- | --- | --- | --- |
| **Cyclosporine** | **Skin cancer** | Jensen, 1999 | 1.24 (1.02-1.52) | 0.032 | 0.06 | 0.24 | 69.5% |
|  |  | Mithoefer, 2002 | 1.25 (1.03-1.51) | 0.027 | 0.06 | 0.24 | 69.0% |
|  |  | Kasiske, 2004 | 1.43 (1.11-1.85) | 0.006 | 0.12 | 0.34 | 73.3% |
|  |  | Molina, 2010 | 1.37 (1.09-1.72) | 0.007 | 0.08 | 0.29 | 74.5% |
|  |  | Ingvar, 2010 | 1.30 (1.05-1.61) | 0.015 | 0.07 | 0.27 | 73.7% |
|  |  | Keller, 2010 | 1.32 (1.07-1.62) | 0.010 | 0.07 | 0.27 | 74.3% |
|  |  | Clarke, 2015 | 1.31 (1.05-1.63) | 0.016 | 0.08 | 0.27 | 73.5% |
|  |  | Coghill, 2016 | 1.36 (1.09-1.70) | 0.006 | 0.08 | 0.28 | 74.4% |
|  |  | Lange, 2016 | 1.26 (1.03-1.55) | 0.025 | 0.06 | 0.25 | 71.4% |
|  |  | Hamandi, 2018 | 1.39 (1.13-1.71) | 0.002 | 0.07 | 0.26 | 71.3% |
|  |  | Bhat, 2018 | 1.44 (1.09-1.92) | 0.011 | 0.15 | 0.39 | 74.5% |
|  |  | Cahoon, 2018 | 1.40 (1.12-1.73) | 0.003 | 0.07 | 0.27 | 72.5% |
|  |  | Tseng, 2018 | 1.23 (1.02-1.48) | 0.032 | 0.05 | 0.22 | **65.8%** |
|  |  |  |  |  |  |  |  |
|  |  | **Pooled estimate** | **1.32 (1.07-1.62)** | **0.008** | **0.07** | **0.27** | **72.2%** |
|  | **Hematologic cancer** | Caillard, 2006 | 0.99 (0.88-1.11) | 0.848 | 0.00 | 0.00 | 0.0% |
|  |  | Fernberg, 2011 | 0.94 (0.83-1.07) | 0.370 | 0.00 | 0.06 | 15.0% |
|  |  | Caillard, 2012 | 0.97 (0.87-1.08) | 0.561 | 0.00 | 0.00 | 0.0% |
|  |  | Na, 2016 | 0.96 (0.86-1.08) | 0.483 | 0.00 | 0.02 | 2.7% |
|  |  | Regan, 2017 | 0.91 (0.79-1.04) | 0.177 | 0.00 | 0.00 | 0.0% |
|  |  | Bhat, 2018 | 0.92 (0.78-1.07) | 0.263 | 0.00 | 0.06 | 7.5% |
|  |  |  |  |  |  |  |  |
|  |  | **Pooled estimate** | **0.96 (0.86-1.07)** | **0.413** | **0.00** | **0.00** | **0.0%** |
| **Azathioprine** | **Skin cancer** | Kasiske, 2004 | 1.66 (1.30-2.13) | <0.0001 | 0.08 | 0.29 | 52.0% |
|  |  | Molina, 2010 | 1.60 (1.25-2.06) | 0.0002 | 0.09 | 0.30 | 60.0% |
|  |  | Ingvar, 2010 | 1.45 (1.20-1.75) | 0.0001 | 0.04 | 0.20 | 46.5% |
|  |  | Keller, 2010 | 1.56 (1.25-1.94) | <0.0001 | 0.07 | 0.27 | 60.9% |
|  |  | Singh, 2011 | 1.61 (1.27-2.04) | <0.0001 | 0.08 | 0.29 | 61.2% |
|  |  | Setshedi, 2012 | 1.51 (1.23-1.86) | 0.0001 | 0.06 | 0.25 | 56.6% |
|  |  | Pasternak, 2013 | 1.56 (1.24-1.96) | 0.0001 | 0.07 | 0.27 | 60.6% |
|  |  | Pedersen, 2014 | 1.47 (1.20-1.81) | 0.0002 | 0.05 | 0.23 | 52.7% |
|  |  | Clarke, 2015 | 1.59 (1.25-2.02) | 0.0002 | 0.08 | 0.29 | 60.6% |
|  |  | Coghill, 2016 | 1.50 (1.21-1.86) | 0.0002 | 0.06 | 0.25 | 56.4% |
|  |  | Lange, 2016 | 1.54 (1.23-1.93) | 0.0001 | 0.07 | 0.27 | 60.0% |
|  |  | Hamandi, 2018 | 1.61 (1.27-2.03) | <0.0001 | 0.08 | 0.28 | 61.1% |
|  |  | Cahoon, 2018 | 1.65 (1.32-2.06) | <0.0001 | 0.06 | 0.25 | 54.6% |
|  |  |  |  |  |  |  |  |
|  |  | **Pooled estimate** | **1.56 (1.25-1.93)** | **<0.0001** | **0.07** | **0.26** | **57.7%** |
|  | **Hematologic cancer** | Caillard, 2006 | 1.68 (1.14-2.49) | 0.009 | 0.31 | 0.56 | 84.6% |
|  |  | Bernatsky, 2008^1^ | 1.62 (1.12-2.35) | 0.011 | 0.27 | 0.52 | 85.1% |
|  |  | Bernatsky, 2008 | 1.56 (1.10-2.20) | 0.012 | 0.23 | 0.48 | 85.1% |
|  |  | Beaugerie, 2009 | 1.39 (1.02-1.91) | 0.040 | 0.19 | 0.43 | 82.2% |
|  |  | Zimmerman, 2010 | 1.45 (1.04-2.01) | 0.026 | 0.21 | 0.46 | 83.9% |
|  |  | Fernberg, 2011 | 1.49 (1.07-2.08) | 0.018 | 0.22 | 0.47 | 84.7% |
|  |  | Caillard, 2012 | 1.56 (1.10-2.23) | 0.014 | 0.24 | 0.49 | 84.9% |
|  |  | Khan, 2013 | 1.28 (0.98-1.69) | 0.074 | 0.12 | 0.34 | 72.6% |
|  |  | Pasternak, 2013 | 1.47 (1.05-2.05) | 0.026 | 0.22 | 0.47 | 84.0% |
|  |  | Mbulaiteye, 2013 | 1.70 (1.21-2.40) | 0.002 | 0.22 | 0.47 | 83.4% |
|  |  | Na, 2016 | 1.50 (1.06-2.11) | 0.022 | 0.23 | 0.48 | 84.2% |
|  |  | Regan, 2017 | 1.69 (1.11-2.59) | 0.015 | 0.38 | 0.62 | 83.1% |
|  |  |  |  |  |  |  |  |
|  |  | **Pooled estimate** | **1.53 (1.10-2.12)** | **0.011** | **0.22** | **0.47** | **83.6%** |
| **Cyclophosphamide** | **Bladder cancer** | Travis, 1995 | 2.20 (0.67-7.20) | 0.191 | 0.42 | 0.65 | 56.8% |
|  |  | Kaldor, 1995 | 2.31 (0.58-9.24) | 0.237 | 0.63 | 0.79 | 62.6% |
|  |  | Xu, 2013 | 4.00 (1.99-8.02) | <0.0001 | 0.00 | 0.00 | 0.0% |
|  |  |  |  |  |  |  |  |
|  |  | **Pooled estimate** | **2.87 (1.32-6.23)** | **0.008** | **0.17** | **0.41** | **35.2%** |
|  | **Hematologic cancer** | Kaldor, 1990 | 2.44 (1.45-4.10) | 0.0008 | 0.18 | 0.43 | 47.0% |
|  |  | Nandakumar, 1991 | 2.13 (1.64-2.78) | <0.0001 | 0.00 | 0.00 | 0.0% |
|  |  | Curtis, 1992 | 2.37 (1.51-3.70) | 0.0002 | 0.13 | 0.36 | 47.2% |
|  |  | Travis, 1994 | 2.56 (1.64-4.00) | <0.0001 | 0.13 | 0.36 | 48.5% |
|  |  | Bernatsky, 2008^1^ | 2.73 (1.67-4.44) | <0.0001 | 0.13 | 0.36 | 37.1% |
|  |  | Bernatsky, 2008 | 2.50 (1.61-3.90) | <0.0001 | 0.13 | 0.37 | 49.7% |
|  |  | Xu, 2013 | 2.56 (1.70-3.86) | <0.0001 | 0.11 | 0.33 | 45.5% |
|  |  |  |  |  |  |  |  |
|  |  | **Pooled estimate** | **2.43 (1.65-3.58)** | **<0.0001** | **0.10** | **0.31** | **39.8%** |
| **Melphalan** | **Hematologic cancer** | Kaldor, 1990 | 7.69 (1.19-49.59) | 0.0319 | 1.52 | 1.23 | 84.1% |
|  |  | Curtis, 1992 | 5.83 (0.56-61.20) | 0.1418 | 2.69 | 1.64 | 93.1% |
|  |  | Travis, 1999 | 2.03 (1.43-2.88) | <0.0001 | 0.01 | 0.08 | 5.3% |
|  |  |  |  |  |  |  |  |
|  |  | **Pooled estimate** | **4.43 (1.30-15.15)** | **0.0176** | **0.99** | **1.00** | **86.5%** |

Abbreviations: RR, relative risk; CI, confidence interval. 1. Cohort study.

Unable to conduct influential meta-analysis on busulfan, chlorambucil, methoxsalen + UV, etoposide, thiotepa and MOPP for having less than 3 studies.

**Identification of studies via databases and registers**

Records removed *before screening*:

Duplicate records removed (n = 0)

Records marked as ineligible by automation tools (n = 0)

Records removed for other reasons (n = 0)

Records identified from:

Databases (n = 2,522)

Registers (n = 0)

**Identification**

Records screened

(n = 2,522)

Records excluded

(n = 2,483)

Reports sought for retrieval

(n = 39)

Reports not retrieved

(n = 0)

**Screening**

Reports assessed for eligibility

(n = 39)

Reports excluded:

Case study (n = 13)

Review article or letter (n = 4)

No effect size (n = 9)

Studies included in review

(n = 13)

**Included**

Supplementary Figure 1. PRISMA flowchart of systematic reviews on cyclosporine and skin cancer.

**Identification of studies via databases and registers**

Records removed *before screening*:

Duplicate records removed (n = 0)

Records marked as ineligible by automation tools (n = 0)

Records removed for other reasons (n = 0)

Records identified from:

Databases (n = 8,444)

Registers (n = 0)

**Identification**

Records screened

(n = 8,444)

Records excluded

(n = 8,338)

Reports sought for retrieval

(n = 106)

Reports not retrieved

(n = 0)

**Screening**

Reports assessed for eligibility

(n = 106)

Reports excluded:

Case study (n = 35)

Review article or letter

(n = 21)

No effect size (n = 44)

Studies included in review

(n = 6)

**Included**

Supplementary Figure 2. PRISMA flowchart of systematic reviews on cyclosporine and hematologic cancer.

**Identification of studies via databases and registers**

Records removed *before screening*:

Duplicate records removed (n = 0)

Records marked as ineligible by automation tools (n = 0)

Records removed for other reasons (n = 0)

Records identified from:

Databases (n = 1,854)

Registers (n = 0)

**Identification**

Records screened

(n = 1,854)

Records excluded

(n = 1,823)

Reports sought for retrieval

(n = 31)

Reports not retrieved

(n = 0)

**Screening**

Reports assessed for eligibility

(n = 31)

Reports excluded:

Case study (n = 11)

Review article or letter

(n = 2)

No effect size (n = 5)

Studies included in review

(n = 13)

**Included**

Supplementary Figure 3. PRISMA flowchart of systematic reviews on azathioprine and skin cancer.

**Identification of studies via databases and registers**

Records removed *before screening*:

Duplicate records removed (n = 0)

Records marked as ineligible by automation tools (n = 0)

Records removed for other reasons (n = 0)

Records identified from:

Databases (n = 4,027)

Registers (n = 0)

**Identification**

Records screened

(n = 4,027)

Records excluded

(n = 3,977)

Reports sought for retrieval

(n = 50)

Reports not retrieved

(n = 0)

**Screening**

Reports assessed for eligibility

(n = 50)

Reports excluded:

Case study (n = 17)

Review article or letter

(n = 5)

No effect size (n = 16)

Studies included in review

(n = 12)

**Included**

Supplementary Figure 4. PRISMA flowchart of systematic reviews on azathioprine and hematologic cancer.

**Identification of studies via databases and registers**

Records removed *before screening*:

Duplicate records removed (n = 0)

Records marked as ineligible by automation tools (n = 0)

Records removed for other reasons (n = 0)

Records identified from:

Databases (n = 1,782)

Registers (n = 0)

**Identification**

Records screened

(n = 1,782)

Records excluded

(n = 1,762)

Reports sought for retrieval

(n = 20)

Reports not retrieved

(n = 0)

**Screening**

Reports assessed for eligibility

(n = 20)

Reports excluded:

Case study (n = 7)

Review article or letter

(n = 2)

No effect size (n = 8)

Studies included in review

(n = 3)

**Included**

Supplementary Figure 5. PRISMA flowchart of systematic reviews on cyclophosphamide and bladder cancer.

**Identification of studies via databases and registers**

Records removed *before screening*:

Duplicate records removed (n = 0)

Records marked as ineligible by automation tools (n = 0)

Records removed for other reasons (n = 0)

Records identified from:

Databases (n = 50,301)

Registers (n = 0)

**Identification**

Records screened

(n = 50,301)

Records excluded

(n = 50,220)

Reports sought for retrieval

(n = 81)

Reports not retrieved

(n = 0)

**Screening**

Reports assessed for eligibility

(n = 81)

Reports excluded:

Case study (n = 43)

Review article or letter

(n = 10)

No effect size (n = 21)

Studies included in review

(n = 7)

**Included**

Supplementary Figure 6. PRISMA flowchart of systematic reviews on cyclophosphamide and hematologic cancer.

**Identification of studies via databases and registers**

Records removed *before screening*:

Duplicate records removed (n = 0)

Records marked as ineligible by automation tools (n = 0)

Records removed for other reasons (n = 0)

Records identified from:

Databases (n = 5,131)

Registers (n = 0)

**Identification**

Records screened

(n = 5,131)

Records excluded

(n = 5,106)

Reports sought for retrieval

(n = 25)

Reports not retrieved

(n = 0)

**Screening**

Reports assessed for eligibility

(n = 25)

Reports excluded:

Case study (n = 12)

Review article or letter

(n = 5)

No effect size (n = 6)

Studies included in review

(n = 2)

**Included**

Supplementary Figure 7. PRISMA flowchart of systematic reviews on busulfan and hematologic cancer.

**Identification of studies via databases and registers**

Records removed *before screening*:

Duplicate records removed (n = 0)

Records marked as ineligible by automation tools (n = 0)

Records removed for other reasons (n = 0)

Records identified from:

Databases (n = 642)

Registers (n = 0)

**Identification**

Records screened

(n = 642)

Records excluded

(n = 617)

Reports sought for retrieval

(n = 25)

Reports not retrieved

(n = 0)

**Screening**

Reports assessed for eligibility

(n = 25)

Reports excluded:

Case study (n = 9)

Review article or letter

(n = 2)

No effect size (n = 13)

Studies included in review

(n = 1)

**Included**

Supplementary Figure 8. PRISMA flowchart of systematic reviews on methoxsalen + UV and skin cancer.

**Identification of studies via databases and registers**

Records removed *before screening*:

Duplicate records removed (n = 0)

Records marked as ineligible by automation tools (n = 0)

Records removed for other reasons (n = 0)

Records identified from:

Databases (n = 8,358)

Registers (n = 0)

**Identification**

Records screened

(n = 8,358)

Records excluded

(n = 8,328)

Reports sought for retrieval

(n = 30)

Reports not retrieved

(n = 0)

**Screening**

Reports assessed for eligibility

(n = 30)

Reports excluded:

Case study (n = 17)

Review article or letter

(n = 4)

No effect size (n = 6)

Studies included in review

(n = 3)

**Included**

Supplementary Figure 9. PRISMA flowchart of systematic reviews on melphalan and hematologic cancer.

**Identification of studies via databases and registers**

Records removed *before screening*:

Duplicate records removed (n = 0)

Records marked as ineligible by automation tools (n = 0)

Records removed for other reasons (n = 0)

Records identified from:

Databases (n = 4,965)

Registers (n = 0)

**Identification**

Records screened

(n = 4,965)

Records excluded

(n = 4,952)

Reports sought for retrieval

(n = 13)

Reports not retrieved

(n = 0)

**Screening**

Reports assessed for eligibility

(n = 13)

Reports excluded:

Case study (n = 7)

Review article or letter

(n = 3)

No effect size (n = 1)

Studies included in review

(n = 2)

**Included**

Supplementary Figure 10. PRISMA flowchart of systematic reviews on chlorambucil and hematologic cancer.

**Identification of studies via databases and registers**

Records removed *before screening*:

Duplicate records removed (n = 0)

Records marked as ineligible by automation tools (n = 0)

Records removed for other reasons (n = 0)

Records identified from:

Databases (n = 1,883)

Registers (n = 0)

**Identification**

Records screened

(n = 1,883)

Records excluded

(n = 1,879)

Reports sought for retrieval

(n = 4)

Reports not retrieved

(n = 0)

**Screening**

Reports assessed for eligibility

(n = 4)

Reports excluded:

Case study (n = 0)

Review article or letter

(n = 0)

No effect size (n = 3)

Studies included in review

(n = 1)

**Included**

Supplementary Figure 11. PRISMA flowchart of systematic reviews on thiotepa and hematologic cancer.

**Identification of studies via databases and registers**

Records removed *before screening*:

Duplicate records removed (n = 0)

Records marked as ineligible by automation tools (n = 0)

Records removed for other reasons (n = 0)

Records identified from:

Databases (n = 59)

Registers (n = 0)

**Identification**

Records screened

(n = 59)

Records excluded

(n = 57)

Reports sought for retrieval

(n = 2)

Reports not retrieved

(n = 0)

**Screening**

Reports assessed for eligibility

(n = 2)

Reports excluded:

Case study (n = 0)

Review article or letter

(n = 0)

No effect size (n = 2)

Studies included in review

(n = 0)

**Included**

Supplementary Figure 12. PRISMA flowchart of systematic reviews on treosulfan and hematologic cancer.

**Identification of studies via databases and registers**

Records removed *before screening*:

Duplicate records removed (n = 0)

Records marked as ineligible by automation tools (n = 0)

Records removed for other reasons (n = 0)

Records identified from:

Databases (n = 677)

Registers (n = 0)

**Identification**

Records screened

(n = 677)

Records excluded

(n = 661)

Reports sought for retrieval

(n = 16)

Reports not retrieved

(n = 0)

**Screening**

Reports assessed for eligibility

(n = 16)

Reports excluded:

Case study (n = 5)

Review article or letter

(n = 2)

No effect size (n = 8)

Studies included in review

(n = 1)

**Included**

Supplementary Figure 13. PRISMA flowchart of systematic reviews on MOPP and lung cancer.

**Identification of studies via databases and registers**

Records removed *before screening*:

Duplicate records removed (n = 0)

Records marked as ineligible by automation tools (n = 0)

Records removed for other reasons (n = 0)

Records identified from:

Databases (n = 445)

Registers (n = 0)

**Identification**

Records screened

(n = 445)

Records excluded

(n = 443)

Reports sought for retrieval

(n = 2)

Reports not retrieved

(n = 0)

**Screening**

Reports assessed for eligibility

(n = 2)

Reports excluded:

Case study (n = 0)

Review article or letter

(n = 0)

No effect size (n = 2)

Studies included in review

(n = 0)

**Included**

Supplementary Figure 14. PRISMA flowchart of systematic reviews on MOPP and hematologic cancer.

**Identification of studies via databases and registers**

Records removed *before screening*:

Duplicate records removed (n = 0)

Records marked as ineligible by automation tools (n = 0)

Records removed for other reasons (n = 0)

Records identified from:

Databases (n = 63)

Registers (n = 0)

**Identification**

Records screened

(n = 63)

Records excluded

(n = 54)

Reports sought for retrieval

(n = 9)

Reports not retrieved

(n = 0)

**Screening**

Reports assessed for eligibility

(n = 9)

Reports excluded:

Case study (n = 1)

Review article or letter

(n = 5)

No effect size (n = 3)

Studies included in review

(n = 0)

**Included**

Supplementary Figure 15. PRISMA flowchart of systematic reviews on BEP and hematologic cancer.

**Identification of studies via databases and registers**

Records removed *before screening*:

Duplicate records removed (n = 0)

Records marked as ineligible by automation tools (n = 0)

Records removed for other reasons (n = 0)

Records identified from:

Databases (n = 4,746)

Registers (n = 0)

**Identification**

Records screened

(n = 4,746)

Records excluded

(n = 4,733)

Reports sought for retrieval

(n = 13)

Reports not retrieved

(n = 0)

**Screening**

Reports assessed for eligibility

(n = 13)

Reports excluded:

Case study (n = 1)

Review article or letter

(n = 5)

No effect size (n = 6)

Studies included in review

(n = 1)

**Included**

Supplementary Figure 16. PRISMA flowchart of systematic reviews on etoposide and hematologic cancer.


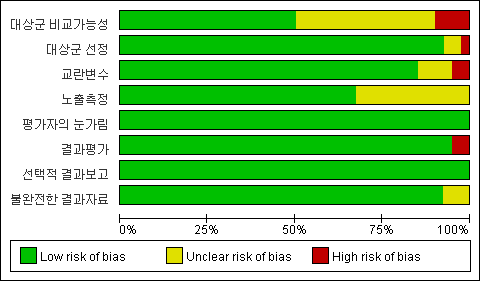

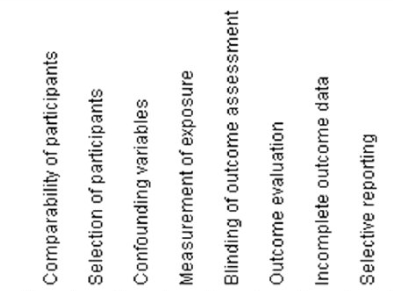

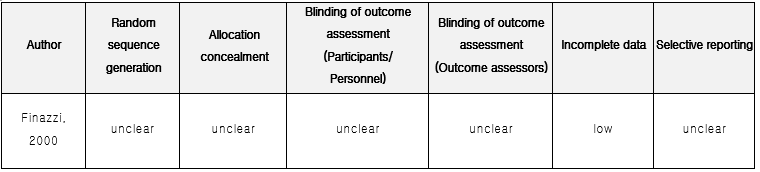


Supplementary Figure 17. Summarized results of the quality assessment for included studies using RoB and RoBANS.


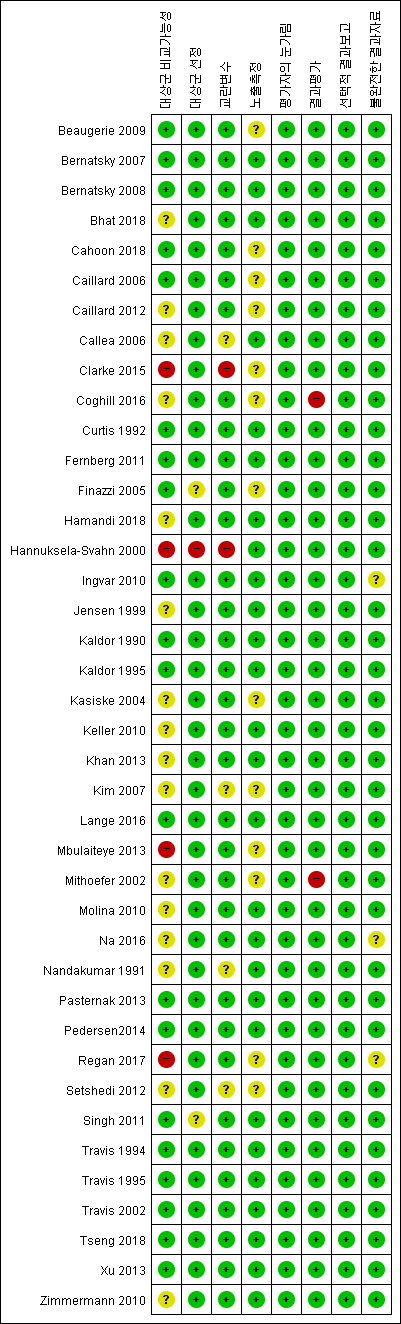

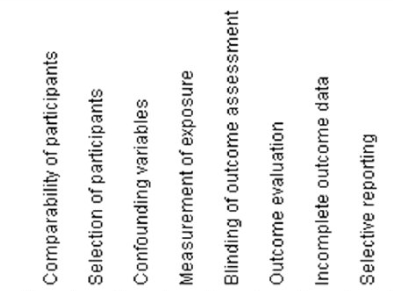

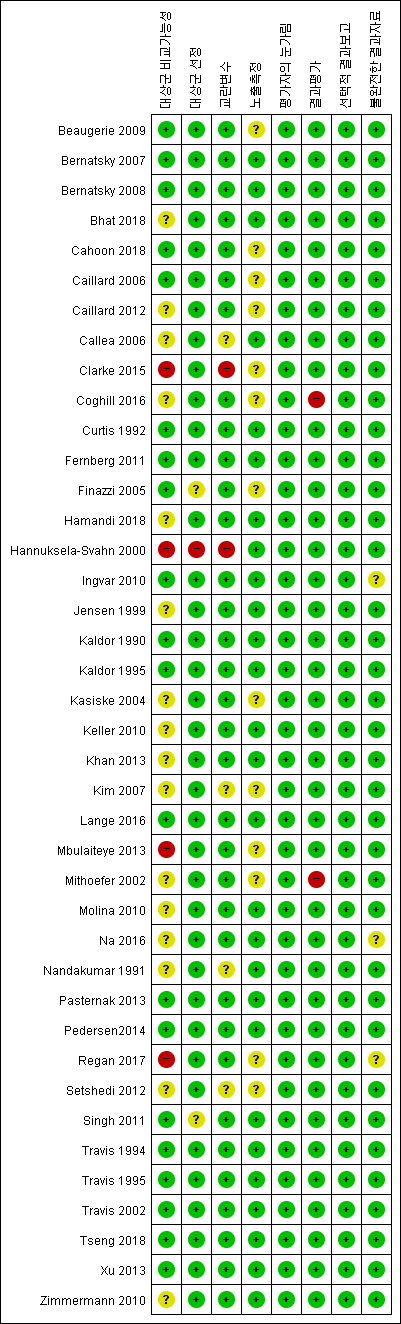

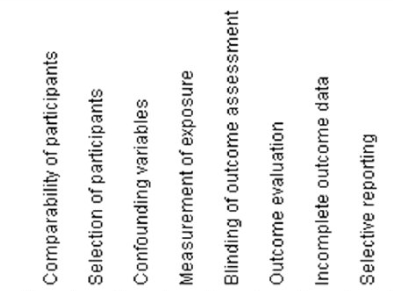

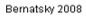

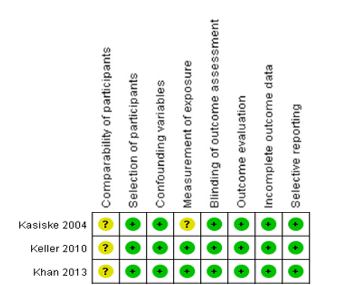

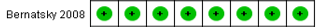

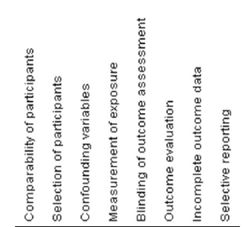

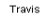

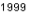


Llea

Supplementary Figure 18. Individual results of the quality assessment for included literature using RoBANS.


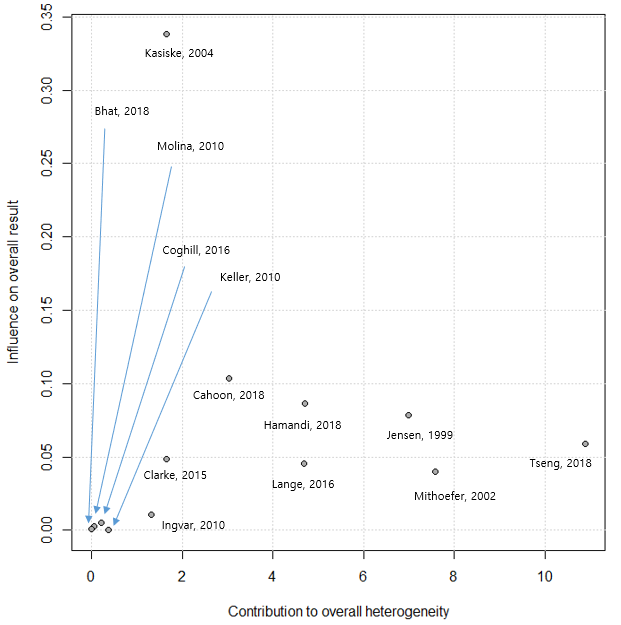


Supplementary Figure 19. Sensitivity analysis: Influence on overall SRR and contribution to overall heterogeneity of studies on association between cyclosporine and skin cancer.


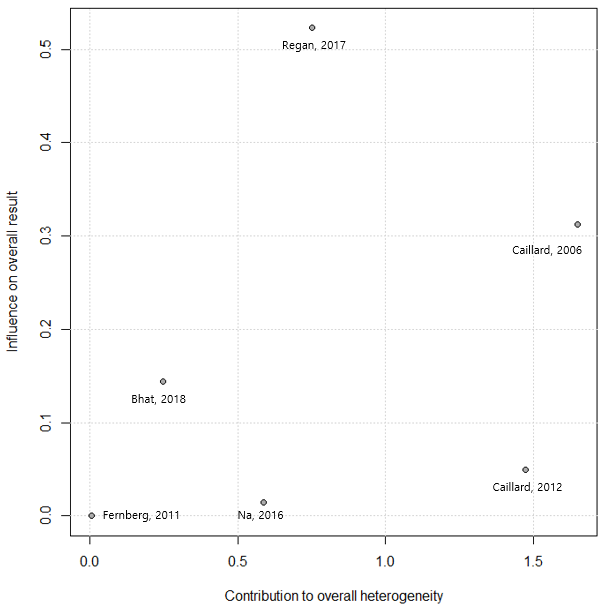


Supplementary Figure 20. Sensitivity analysis: Influence on overall SRR and contribution to overall heterogeneity of studies on association between cyclosporine and hematologic cancer.


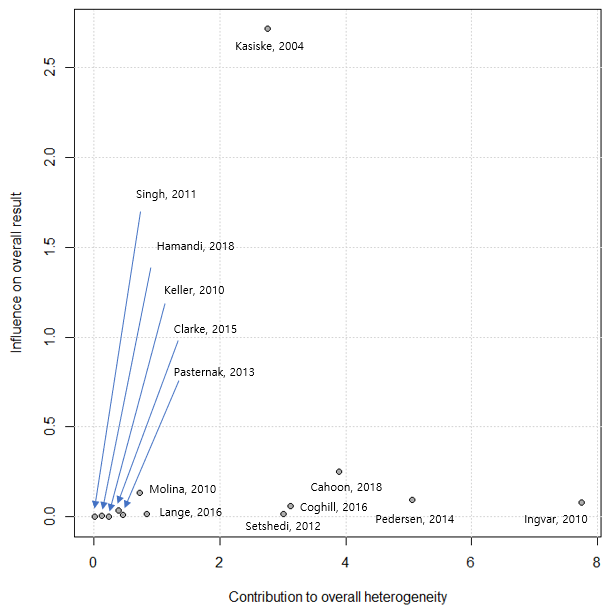


Supplementary Figure 21. Sensitivity analysis: Influence on overall SRR and contribution to overall heterogeneity of studies on association between azathioprine and skin cancer.


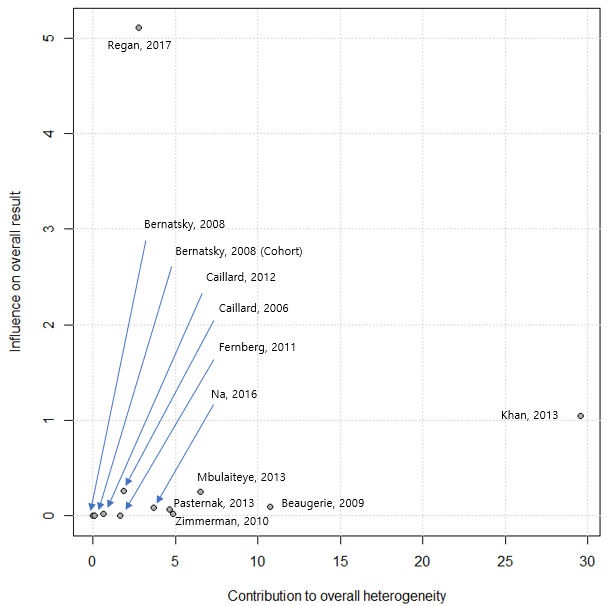


Supplementary Figure 22. Sensitivity analysis: Influence on overall SRR and contribution to overall heterogeneity of studies on association between azathioprine and hematologic cancer.


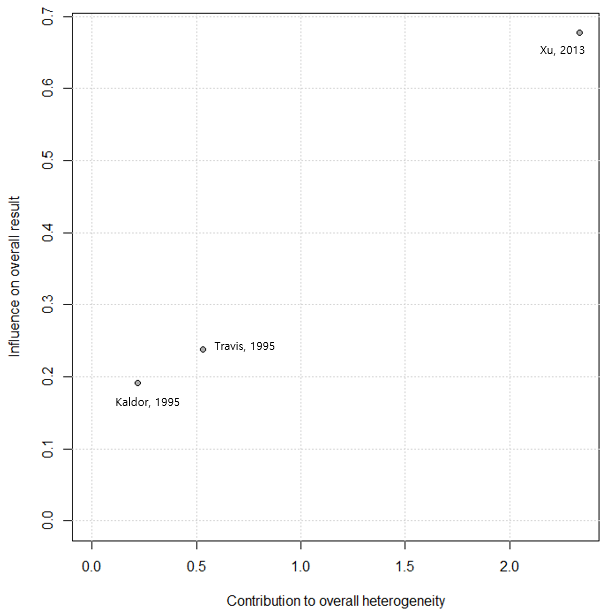


Supplementary Figure 23. Sensitivity analysis: Influence on overall SRR and contribution to overall heterogeneity of studies on association between cyclophosphamide and bladder cancer.


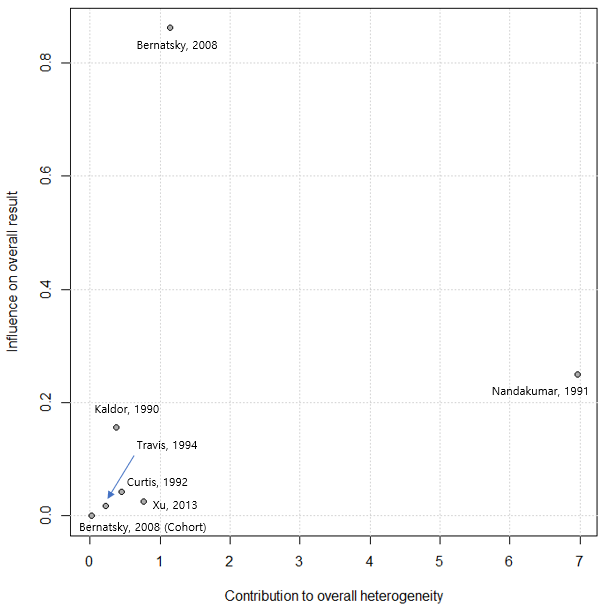


Supplementary Figure 24. Sensitivity analysis: Influence on overall SRR and contribution to overall heterogeneity of studies on association between cyclophosphamide and hematologic cancer.


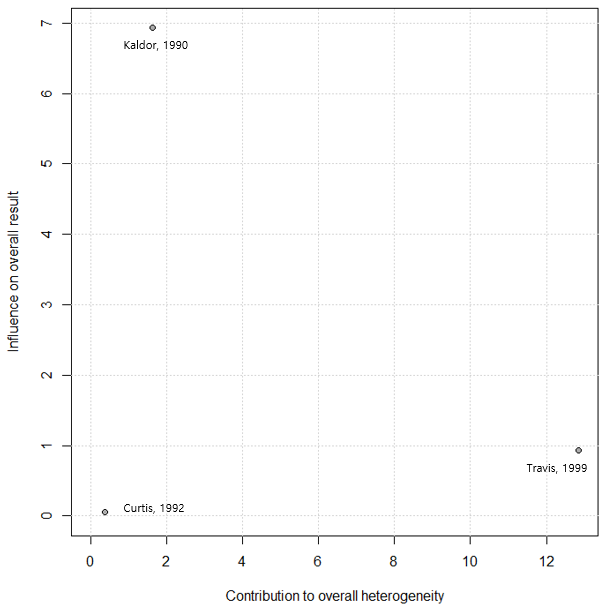


Supplementary Figure 25. Sensitivity analysis: Influence on overall SRR and contribution to overall heterogeneity of studies on association between melphalan and hematologic cancer.
